# CDC42 controls the activation of primordial follicles by regulating PI3K signaling in mouse oocytes

**DOI:** 10.1186/s12915-018-0541-4

**Published:** 2018-07-05

**Authors:** Hao Yan, Jiawei Zhang, Jia Wen, Yibo Wang, Wanbao Niu, Zhen Teng, Tongtong Zhao, Yanli Dai, Yan Zhang, Chao Wang, Yingying Qin, Guoliang Xia, Hua Zhang

**Affiliations:** 1 State Key Laboratory of Agrobiotechnology, College of Biological Science, China Agricultural University, Beijing, 100193 China; 2 Center for Reproductive Medicine, Shandong Provincial Hospital Affiliated to Shandong University, Jinan, 250021 China

**Keywords:** CDC42, PI3K signaling, Primordial follicle activation, In vitro activation, Premature ovarian insufficiency

## Abstract

**Background:**

In mammalian females, progressive activation of dormant primordial follicles in adulthood is crucial for the maintenance of the reproductive lifespan. Misregulated activation of primordial follicles leads to various ovarian diseases, such as premature ovarian insufficiency (POI). Although recent studies have revealed that several functional genes and pathways, such as phosphoinositide 3-kinase (PI3K) signaling, play roles in controlling the activation of primordial follicles, our understanding of the molecular networks regulating the activation progress is still incomplete.

**Results:**

Here, we identify a new role for cell division cycle 42 (CDC42) in regulating the activation of primordial follicles in mice. Our results show that CDC42 expression increases in oocytes during the activation of primordial follicles in the ovary. Disruption of CDC42 activity with specific inhibitors or knockdown of *Cdc42* expression significantly suppresses primordial follicle activation in cultured mouse ovaries. Conversely, the follicle activation ratio is remarkably increased by overexpression of CDC42 in ovaries. We further demonstrate that CDC42 governs the process of primordial follicle activation by binding to phosphatidylinositol-4,5-bisphosphate 3-kinase catalytic subunit beta (p110β) and regulating the expression levels of PTEN in oocytes. Finally, we extend our study to potential clinical applications and show that a short-term in vitro treatment with CDC42 activators could significantly increase the activation rates of primordial follicles in both neonatal and adult mouse ovaries.

**Conclusion:**

Our results reveal that CDC42 controls the activation of primordial follicles in the mammalian ovary and that increasing the activity of CDC42 with specific activators might improve the efficiency of in vitro activation approaches, opening avenues for infertility treatments.

**Electronic supplementary material:**

The online version of this article (10.1186/s12915-018-0541-4) contains supplementary material, which is available to authorized users.

## Background

The non-renewal ovarian follicles serve as the only source of fertilizable ova in female mammals [1]. As the initial stage of follicle development, primordial follicles which the number is established in early life decide the length of the female reproductive lifespan. A primordial follicle consists of a dormant oocyte and several surrounding pre-granulosa cells (preGCs). To generate mature eggs in the ovary, only a limited number of primordial follicles are progressively recruited into the growing pool through a process called follicle activation [2]. The balance between dormancy and activation of primordial follicles maintains a proper reproductive lifespan in organisms. Disorders in primordial follicle activation might lead to various ovarian diseases such as premature ovarian insufficiency (POI) [3].

The activation of primordial follicles is a complex but orchestrated process [2]. With the development of genetically modified mouse models, our understanding of the mechanisms in regulating primordial follicle activation has greatly improved in the past decade. Several signaling pathways in both oocytes and preGCs have been revealed to have a functional role in controlling primordial follicle activation, such as phosphoinositide 3-kinase (PI3K) signaling in oocytes and mTORC1 signaling in preGCs [4, 5]. Of these pathways, PI3K signaling is identified as the dominant signaling pathway in oocytes that governs the activation and survival of primordial follicles [6]. Ablation of downstream PI3K signaling molecules in oocytes (such as PDK1 or FOXO3a) leads to abnormal development of primordial follicles and POI in adult ovaries [7–9]. Deletion of phosphatase and tensin homolog deleted on chromosome 10 (PTEN), the major suppressor of PI3K signaling, causes the premature activation and depletion of primordial follicles in mice [5]. Although the role of the PI3K pathway in controlling primordial follicle activation has been revealed, the upstream molecules that regulate PI3K activity in dormant oocytes have not been well identified.

With the advanced understanding of the inner mechanisms of follicle activation, awakening primordial follicles in vitro have been practiced in clinical treatment to overcome female infertility [10]. By in vitro stimulating PI3K signaling in oocytes with PTEN inhibitors, a new infertility treatment named in vitro activation (IVA) has been developed, and healthy babies have been born from POI patients using these approaches [11, 12]. As is the case with any technology in its nascent stage, however, the efficiency of IVA is still not satisfactory. Therefore, identification of novel pathways or targets that regulate the activation of primordial follicles is not only important to deepen our understanding of female reproduction but also clinically valuable in order to improve IVA efficiency and simplify the operation process, which will be of great benefit to POI patients.

CDC42, a member of the Rho GTPases family, serves as a key regulator in controlling multi-cellular functions, including actin cytoskeletal dynamics, membrane trafficking, transcription, and cell cycle control [13–15]. Interestingly, previous studies revealed that the intracellular membrane localization of CDC42 excludes PTEN from the cytomembrane, further activating the PI3K pathway in cells [16–18]. Meanwhile, it has been reported that the generation of PIP3, the major product of PI3K signaling, is also related to the cellular localization and the activity of CDC42 [19, 20]. These results indicated that CDC42 might be an upstream regulator of PI3K signaling in cells. In female reproductive development, Wang et al. reported that deletion of CDC42 in oocytes of growing follicles disrupts the maturation of oocytes and leads to female infertility in mice, demonstrating that CDC42 plays a functional role in regulating the development of follicles at their late developmental stage [21]. Most recently, Akera et al. revealed that CDC42 signaling from the oocyte cortex regulates the spindle asymmetry which leads to non-Mendelian chromosome segregation in meiosis [22]. These results showed that CDC42 is a pivotal regulator in oocyte maturation. However, the role of CDC42 in primordial follicle activation has not been described until now.

In the current study, we investigated the functional role of CDC42 in primordial follicle activation. Our results show that oocyte membrane-localized CDC42 controls follicle activation by regulating the activity of PI3K signaling. This is achieved through binding of CDC42 active form to phosphatidylinositol-4,5-bisphosphate 3-kinase catalytic subunit beta (PIK3CB, also called p110β) and regulating PTEN expression in the ovaries. We also reveal that inhibition of CDC42 activity or knockdown of *Cdc42* in ovaries suppresses the activation of primordial follicles. Further, overexpression of CDC42 increases the activation and growth of primordial follicles in mouse ovaries. Finally, we find that a short-term treatment with a CDC42 activator in vitro can significantly increase the activation of primordial follicles in both neonatal and adult mouse ovaries, indicating that CDC42 might be a highly efficient target for the improvement of IVA.

## Results

### Oocyte-expressing CDC42 plays a regulatory role in the activation of primordial follicles

**Fig. 1:**
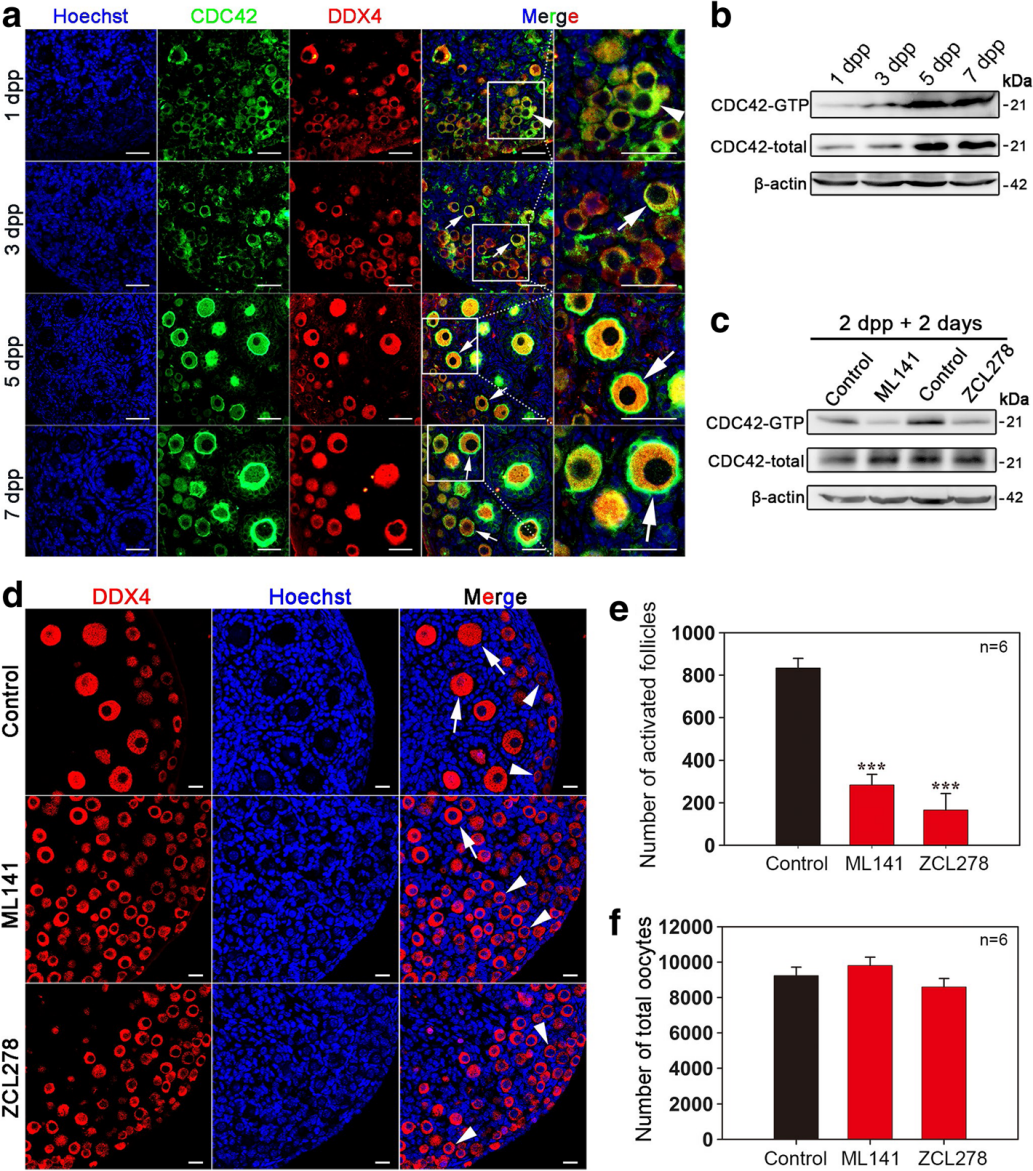
Oocyte-expressed CDC42 regulates the activation of primordial follicles in neonatal mouse ovaries. **a** Cellular localization of CDC42 in perinatal ovaries. Ovaries were stained for CDC42 (green) and the oocyte marker DDX4 (red) at the indicated time points. Nuclei were counter-stained by Hoechst (blue). CDC42 mainly localized to the intracellular membrane of the activated oocytes (arrows). **b** The total protein levels and active form of CDC42 (CDC42-GTP) from 1 to 7 dpp ovaries. Western blot and CDC42-GTP pull-down assays showed that both total and CDC42-GTP expression significantly increased in ovaries at 5 dpp. **c** CDC42-GTP pull-down assay showed that both ML141 and ZCL278 could significantly suppress the expression of CDC42-GTP in culture. **d** Ovaries at 2 dpp were cultured in media alone (control) or with CDC42 inhibitor ML141 or ZCL278 for 5 days in vitro. Oocytes were stained with DDX4 (red). Nuclei were dyed with a Hoechst counter-stain (blue). The activation of oocytes was remarkably suppressed in ML141 or ZCL278-treated ovaries, and few activated oocytes were observed in these ovaries compared to the control. **e**, **f** Quantification of ovarian follicles in cultured ovaries with different treatments. The number of activated follicles significantly decreased in cultured ovaries after ML141 or ZCL27 treatment, and the total number of oocytes was comparable in cultured and treated ovaries (Additional file 10: Individual data values). The asterisks indicate a significant difference between control and treated ovaries. The experiments were repeated at least three times, and representative images are shown. * *P* < 0.05, *** *P* < 0.001. Data are presented as the means ± S.D. Scale bars: (**a**) 50 μm, (**d**) 20 μm

To investigate the function of CDC42 in early follicular development, immunofluorescent staining and Western blot assays were employed to detect the cellular localization and expressing dynamics of CDC42 in perinatal ovaries. CDC42 was mainly detected in the cytoplasm of oocytes in the 1 dpp (day post partum) ovary (Fig. 1a, arrowheads), which contains no activated follicles. Along with ovarian development, CDC42 was consistently expressing in oocytes of follicles at 3, 5, and 7 dpp. Interestingly, high CDC42 expression was observed in the inner side of oocyte membrane in activated follicles (Fig. 1a, arrows). Western blot and CDC42-GTP pull-down assay results revealed that the expression of both CDC42 and its active form, CDC42-GTP, significantly increased with the presence of activated follicles in 5 dpp ovaries (Fig. 1b), indicating that CDC42 might play a regulatory role in the activation of primordial follicles.

We next studied whether CDC42 is involved in the process of primordial follicle activation in ovaries. The first wave of primordial follicles is activated in mouse ovary around 3 dpp, which makes neonatal ovaries an ideal model to study the onset of primordial follicle activation [23]. Therefore, an in vitro neonatal mouse ovarian culture system was established in our study (Additional file 1: Figure S1a). Follicle counting results showed that a comparable number of both total follicles (Additional file 1: Figure S1b) and activated follicles (Additional file 1: Figure S1c) was observed in cultured ovaries and in vivo ovaries at 2 and 7 dpp. These results show that the in vitro system can successfully mimic physiological follicle development in neonatal ovaries. We next cultured 2 dpp ovaries with two different CDC42-selective inhibitors (ML141 or ZCL278). After 48 h of culture, a CDC42-GTP pull-down assay demonstrated that both inhibitors could significantly reduce CDC42-GTP levels in vitro (Fig. 1c), indicating a successful suppression of CDC42 activity in the ovaries. To investigate the functional role of CDC42, ovaries from mice at 2 dpp were cultured for 5 days (equal to 7 dpp in vivo) with or without inhibitors, and the developmental profile of ovaries was investigated by histological detection and follicle number counting. Morphological analyses showed a remarkable suppression of primordial follicle activation after treatment with the CDC42 inhibitor (Fig. 1d, Additional file 2: Figure S2a). Meanwhile, the number of activated follicles in the inhibitor-treated groups (ML141, 290.0 ± 47.3 and ZCL278, 151.7 ± 53.6) was significantly decreased compared with that in control ovaries (839.2 ± 47.5) (Fig. 1e), whereas the total number of oocytes was comparable across all groups (Fig. 1f). These results indicate that CDC42 plays a regulatory role in the process of follicle activation in mice.

### Manipulating the expression of *Cdc42* regulates the activation of primordial follicles

**Fig. 2:**
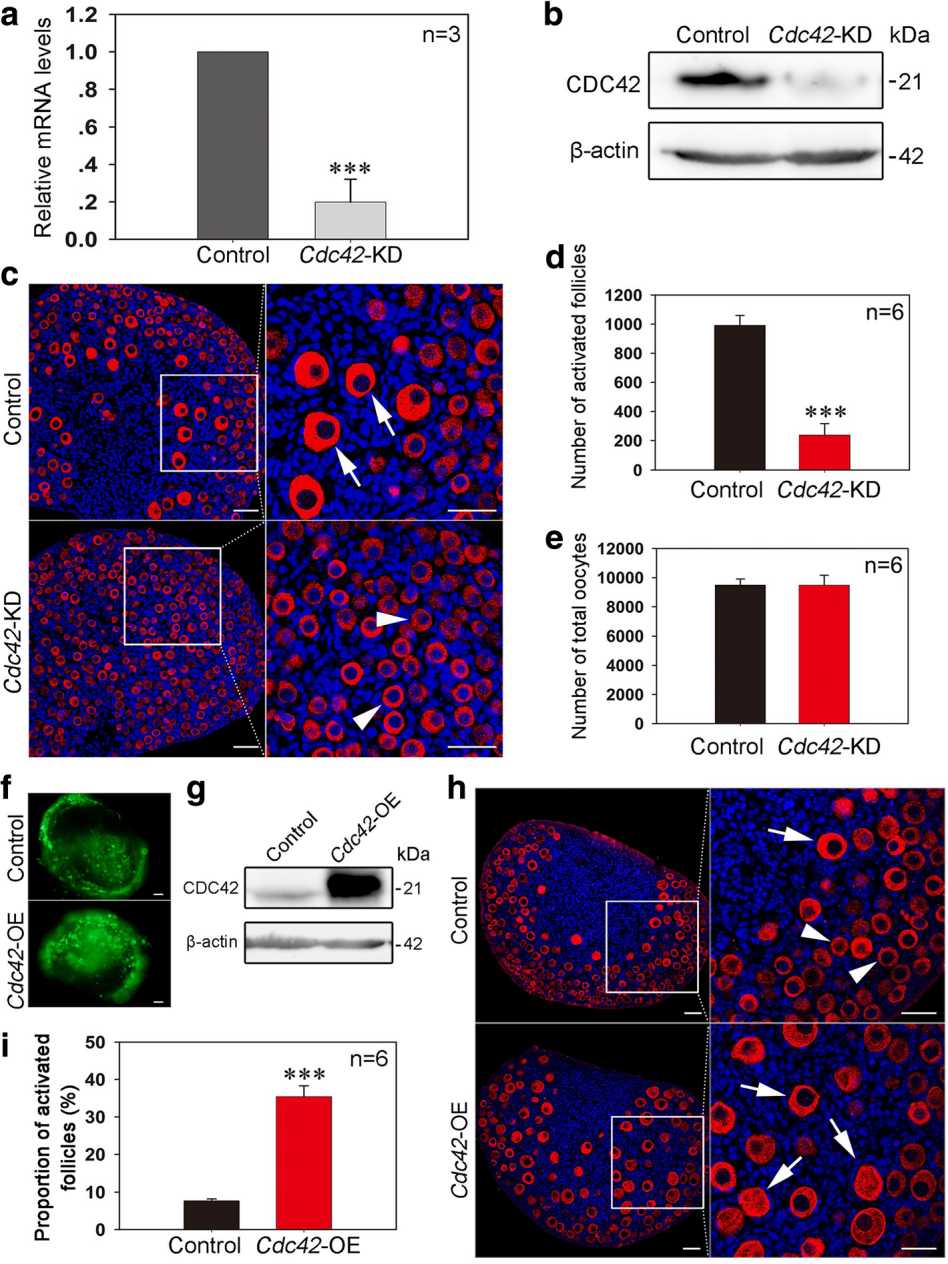
Manipulating the expression of *Cdc42* controls the activation of primordial follicles. **a**, **b** Validating the efficiency of *Cdc42* knockdown in cultured ovaries. Ovaries at 1 dpp were injected with *Cdc42* esiRNA or scrambled siRNA and cultured in vitro for 24 or 48 h. *Cdc42* mRNA and protein expression was significantly decreased in the *Cdc42-*KD group. **c** After *Cdc42* esiRNA injection at 1 dpp, the transfected ovaries were cultured for six more days. Histological analysis showed a normal follicle distribution with activated follicles (arrows) in control ovaries. However, numerous primordial follicles (arrowheads) and few activated follicles were observed in the *Cdc42-*KD ovaries. **d**, **e** Follicle quantification in cultured ovaries showed that knockdown of *Cdc42* significantly inhibited the activation of primordial follicles (*P* < 0.001) but had no effect on the survival of oocytes (Additional file 10: individual data values). **f** The ovaries at 1 dpp were transfected with empty lentivirus or a lentiviral construct expressing *Cdc42* (*Cdc42-*OE) for 2 or 6 days in vitro. Green fluorescence from the GFP reporter was observed in ovaries following 2 days of lentiviral infection, demonstrating a satisfactory efficiency of *Cdc42*-OE. **g** Western blot showed that CDC42 expression was obviously upregulated in *Cdc42*-OE ovaries after 2 days of culture. **h** Morphological analysis showed that ovaries in the *Cdc42*-OE group exhibited more activated follicles (arrows) than the control. Ovaries were stained for the oocyte marker DDX4 (red). Nuclei were dyed with a Hoechst counter-stain (blue). **i** Quantification of ovarian follicles showed a significant increase of activated follicles (34.43 ± 2.34%) in *Cdc42*-OE ovaries compared to controls (8.06 ± 0.80%) (Additional file 10: individual data values). The experiments were repeated at least three times, and representative images are shown. Scale bars, 50 μm

To confirm the role of CDC42 in regulating primordial follicle activation, esiRNA-mediated knockdown of *Cdc42* expression (*Cdc42-*KD) was employed in the cultured neonatal ovary. *Cdc42* esiRNA or a scrambled control was transfected into cultured 1 dpp ovaries. Real-time PCR and Western blot analyses revealed an obvious decrease in *Cdc42* mRNA (Fig. 2a) and protein expression (Fig. 2b) in the *Cdc42*-KD group after 24 or 48 h of culture. The treated ovaries were cultured for five more days (equal to 7 dpp in vivo) and subsequent histological analysis showed a dramatic suppression of primordial follicle activation in *Cdc42-*KD ovaries (Fig. 2c). The total oocytes and activated follicles were counted to determine the role of CDC42 in follicle development. An identical number of total follicles was observed in control and *Cdc42-*KD ovaries, indicating that *Cdc42* knockdown has no effect on oocyte survival and follicle formation. However, a significant decrease in activated follicles was found in *Cdc42-*KD ovaries (235.0 ± 51.5) compared to control ovaries (961.7 ± 57.3) (Fig. 2d, e, Additional file 2: Figure S2b). Therefore, these results demonstrate that the expression of CDC42 is essential in controlling the activation of primordial follicles in the neonatal mouse ovary.

Given that CDC42 is highly expressed in the oocytes of activated follicles, we next examined if increasing CDC42 expression could stimulate the activation of primordial follicles in culture. A lentiviral construct expressing *Cdc42* (*Cdc42*-OE) with a GFP reporter was designed and transfected into 1 dpp ovaries in culture. After 2 days of *Cdc42*-OE treatment, GFP was observed in the entire ovary, indicating a successful transfection in culture (Fig. 2f). Western blot showed that CDC42 expression was significantly increased in *Cdc42*-OE ovaries compared to controls (Fig. 2g). The transfected ovaries were further cultured for 4 days (equal to 7 dpp in vivo), and subsequent morphological analysis revealed that a premature activation of primordial follicles (Fig. 2h, arrows) occurred in *Cdc42*-OE ovaries, which is in sharp contrast to the normal follicle distribution in the control (Fig. 2h, arrowheads). Statistical analysis showed a remarkably larger proportion of activated follicles in *Cdc42*-OE groups compared with control groups (Fig. 2i, Additional file 2: Figure S2c, 34.43 ± 2.34% vs. 8.06 ± 0.80%). These results show that elevating CDC42 expression could increase the activation of primordial follicles in ovaries.

### CDC42 governs follicle activation by controlling the PI3K pathway in ovaries

**Fig. 3:**
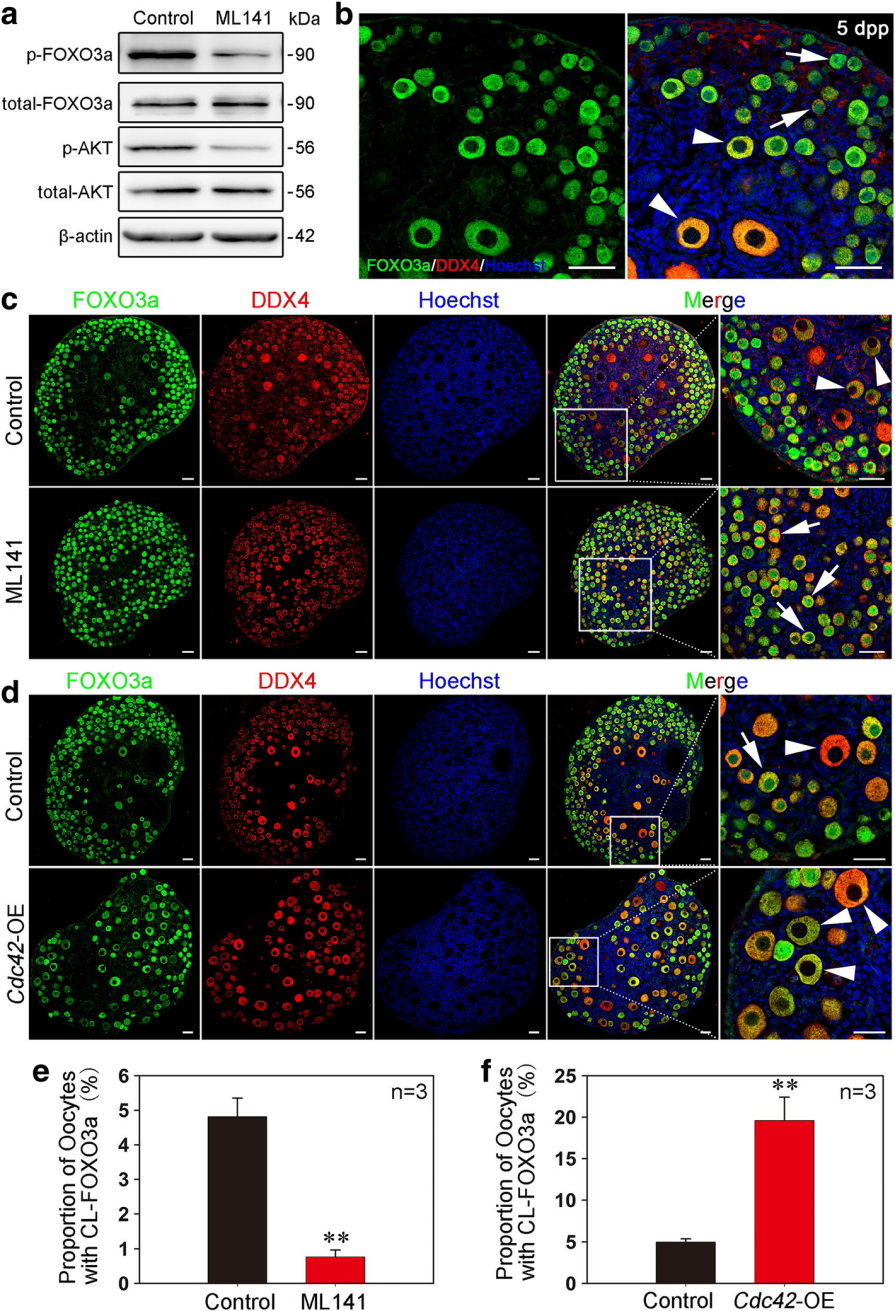
CDC42 in oocytes regulates the PI3K pathway to control follicle activation in neonatal ovaries. **a** Western blot showed that the activity of PI3K signaling was suppressed in ML141-treated ovaries. Levels of total FOXO3a, AKT, and β-actin were used as internal controls. The phosphorylation of FOXO3a and AKT was decreased in ML141-treated ovaries. **b** FOXO3a localizes to the nuclei (arrows) of dormant oocytes and to the cytoplasm (arrowheads) of activated oocytes when PI3K signaling is activated in 5 dpp ovaries. **c** Nuclear localization of FOXO3a (green fluorescence) was observed in oocytes of ML141-treated ovaries indicating suppressed PI3K signaling in oocytes of primordial follicles. Oocytes were stained with DDX4 (red). Nuclei were dyed with a Hoechst counter-stain (blue). **d** Overexpression of CDC42 led to premature follicle activation in ovaries. Cytoplasmic localization of FOXO3a (arrowheads, CL-FOXO3a) was observed in most of the oocytes. **e**, **f** The oocytes with CL-FOXO3a localization were quantified in ML141, *Cdc42*-OE and control ovaries. The proportion of CL-FOXO3a was significantly decreased (*P* < 0.005) in ML141-cultured ovaries but increased (*P* < 0.05) in *Cdc42*-OE-injected ovaries (Additional file 10: individual data values). The experiments were repeated at least three times, and representative images are shown. Scale bars, 40 μm

To further elucidate the underlying mechanisms of how CDC42 controls the activation of primordial follicles, we investigated the relationship between CDC42 and PI3K signaling, the dominant pathway in oocytes that governs the activation of primordial follicles [2, 24]. The expression levels of AKT and FOXO3a, which are independent downstream molecules involved in PI3K signaling, were detected in ovarian lysates of ML141-treated ovaries. Western blot analysis showed a dramatic reduction of p-AKT and p-FOXO3a expression in treated ovaries compared to controls (Fig. 3a), indicating an activating role of CDC42 in the regulation of PI3K signaling in dormant oocytes.

To confirm the role of CDC42 in regulating PI3K signaling activity, we next detected the localization of FOXO3a, which shuttles from the nucleus (Fig. 3b, arrows) to the cytoplasm (Fig. 3b, arrowheads) in mouse oocytes when PI3K signaling is activated. After 5 days of ML141 treatment starting at 2 dpp, only 0.76 ± 0.20% of oocytes in the medulla region displayed cytoplasmic localization of FOXO3a (CL-FOXO3a) (Fig. 3c, e, arrows), which is in sharp contrast to the 4.81 ± 0.54% of CL-FOXO3a oocytes in the control (Fig. 3c, e, arrowheads). In contrast, quantification of oocytes in *Cdc42*-OE groups showed a significant increase in CL-FOXO3a oocytes compared to control (Fig. 3d, f, 19.95 ± 2.59% vs. 4.99 ± 0.40%). To verify the effect of CDC42 in adult life, mouse ovaries at 35 dpp were collected and fragmented in 4 equal parts then cultured with ML141 for 12 h. Immunoblotting results showed that the expression of both p-AKT and p-FOXO3a was remarkable suppressed in treated ovaries compared with the control (Additional file 3: Figure S3a), and nuclear localization of FOXO3a was observed in most of oocytes in primordial follicles after ML141 treatment (Additional file 3: Figure S3b). These data showed that CDC42 plays the same role in regulating the PI3K signaling in adult ovaries. Therefore, our results indicate that CDC42 regulates the activation of primordial follicles by manipulating the activity of PI3K signaling in oocytes.

### CDC42 regulates the ovarian PI3K signaling by binding p110β in oocytes

**Fig. 4:**
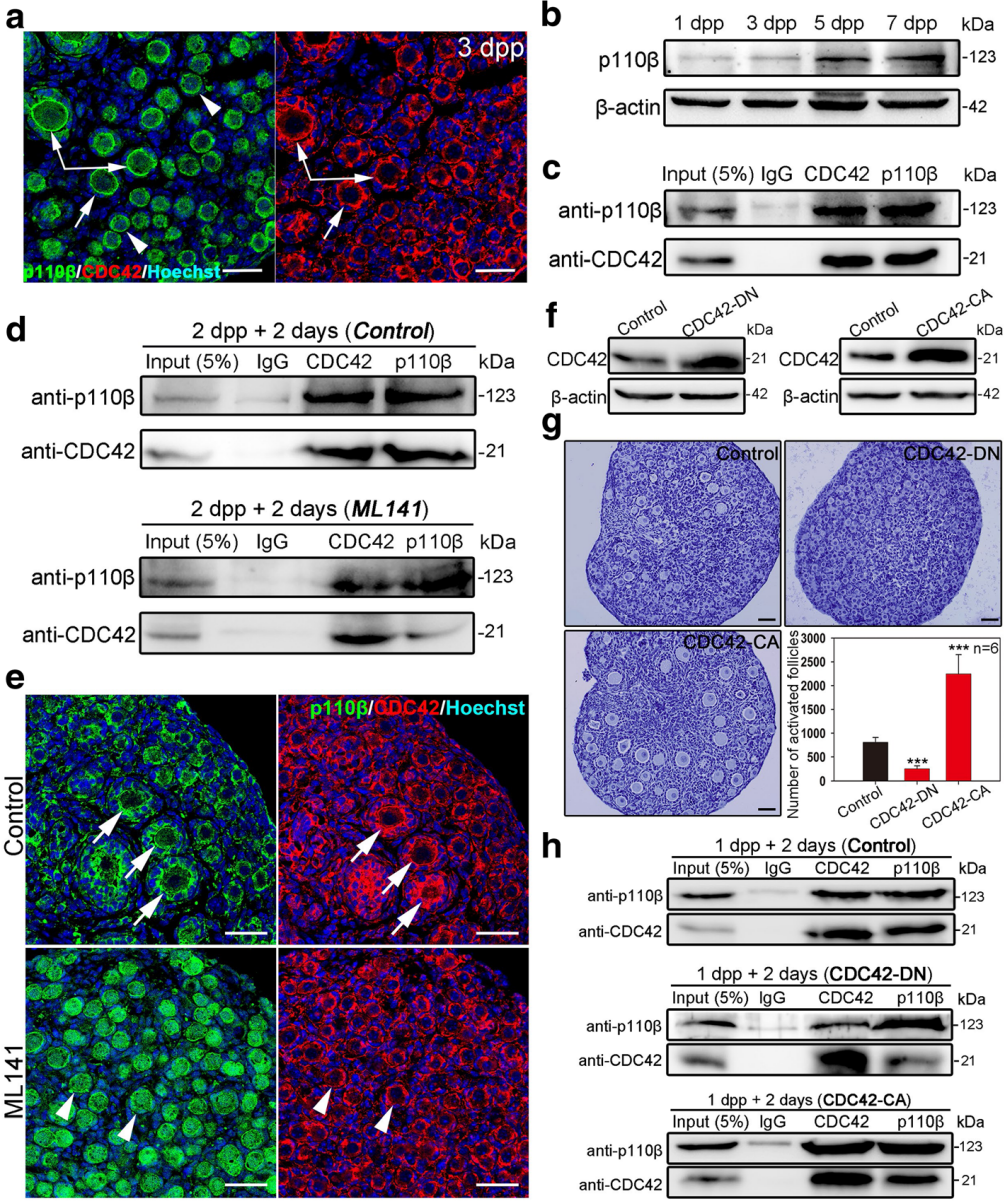
CDC42 regulates PI3K signaling by binding to p110β in activated oocytes. **a** Ovaries at 3 dpp were stained for p110β (green) or CDC42 (red). Nuclei were dyed with a Hoechst (blue). Co-localization of p110β and CDC42 expression was observed in dormant oocytes (arrowheads) and on the intracellular membrane of activated oocytes (arrows). **b** Western blot showed that the expression of p110β increased in 5 and 7 dpp ovaries. **c** The interaction between CDC42 and p110β was detected in ovaries by Co-IP. The protein was extracted from 70 ovaries at 4 dpp. Approximately 5% of lysate was loaded as input. **d** The relationship between the activity of CDC42 and the interaction of CDC42 with p110β. The binding efficiency of CDC42 and p110β was significantly weakened in cultured 2 dpp ovaries after ML141 treatment. **e** Suppressing the activity of CDC42 with ML141 inhibited the intracellular localization of p110β in oocytes. A normal localization of CDC42 and p110β was observed in controls (arrows), while a cytoplasm localization of p110β was found in ML141 treated ovaries (arrowheads). **f** Ovaries at 1 dpp were transfected CDC42-DN or CDC42-CA lentiviral constructs for 2 days; empty lentivirus was used as control. Western blot showed that CDC42 expression was upregulated in ovaries after CDC42-DN or CDC42-CA transfections. **g** Quantification of ovarian follicles showed a decrease of activation (235.0 ± 53.9) in CDC42-DN ovaries and an increased activation (2245.0 ± 361.0) in CDC42-CA ovaries compared to controls (785.8 ± 93.5). **h** The interaction between CDC42 and p110β was examined in CDC42-DN and CDC42-CA treatment by Co-IP. The binding efficiency of CDC42 and p110β was significantly weakened in the CDC42-DN group but improved in the CDC42-CA group. The experiments were repeated at least three times, and representative images are shown. Scale bars, 50 μm

A previous study reported that CDC42 regulates PI3K signaling through interactions with p110β in cultured cells [25]. We therefore examined the relationship between CDC42 and p110β in ovaries in our study. We first measured the localization of p110β in the neonatal ovaries by immunostaining. The results showed that p110β localized to both the cytoplasm and the intracellular membrane of oocytes (Additional file 4: Figure S4a, arrows). Interestingly, high p110β membrane localization was observed in the oocytes of activated follicles, which is correlated with the expression pattern of CDC42 in oocytes (Fig. 4a, arrows). Western blot results showed that p110β expression increased from 5 dpp in ovaries with the activation of primordial follicles (Fig. 4b), indicating a functional role of p110β in follicle activation. Co-immunoprecipitation showed that CDC42 could bind p110β in neonatal ovaries, and showed that CDC42 might be directly regulating PI3K signaling by binding and activating p110β (Fig. 4c). To further investigate how CDC42 regulates p110β in follicle activation, we detected the expression of p110β and the binding efficiency of CDC42 and p110β in ovaries in which the activity of CDC42 was blocked by ML141. Although suppression of CDC42 activity did not affect the expression of p110β in ovaries (Additional file 4: Figure S4b), we found that ML141 treatment noticeably weakened the binding efficiency of CDC42 and p110β compared with the control (Fig. 4d). Meanwhile, the intracellular localization of p110β was depleted in ML141-treated ovaries (Fig. 4e, arrowheads). These results indicate that the binding efficiency of CDC42 and p110β is related to the activity of CDC42 in oocytes.

To further verify the interaction and the inner mechanisms between CDC42 and p110β in follicle activation, two CDC42 mutants, the dominant-negative GDP-bound CDC42T17N (CDC42-DN) and the constitutively activation of the GTP-bound CDC42Q61L (CDC42-CA) [22, 26, 27], were introduced into in vitro ovarian culture system. Lentivirus-CDC42 mutants with GFP reporter were designed and transfected into 1 dpp ovaries in culture, and empty lentivirus was used as the control. After 2 days of treatment, high expression of GFP (Additional file 5: Figure S5a) and increased total CDC42 expression (Fig. 4f) were observed in both CDC42-DN and CDC42-CA ovaries compared to controls, indicating the successful transfections in culture. Follicle counting results showed that the transfection has no effect on survival of oocytes after 6 days of treatment in culture (Additional file 5: Figure S5b). However, the number of activated follicle was significantly decreased in CDC42-DN groups (235.0 ± 53.9), and a significant increase of activated follicles was found in CDC42-CA groups (2245.0 ± 361.0) compared with the control (785.8 ± 93.5) (Fig. 4g). Immunoblotting of p-AKT and FOXO3a staining results showed that a suppressing effect of CDC42-DN and a stimulating effect of CDC42-CA in PI3K signaling activity in treated ovaries (Additional file 5: Figure S5c, S5d), which is consistent with the phenotypes of follicle development. These results confirmed our finding that the activity of CDC42 regulates the activation of primordial follicles through PI3K signaling. We next detected the binding efficiency of CDC42 with p110β in CDC42-DN and CDC42-CA transfected ovaries by co-immunoprecipitation. Although our results showed that the expression of total CDC42 was increased in CDC42-DN ovaries, we found that CDC42-DN remarkably weakened the binding efficiency of CDC42 and p110β compared with the control (Fig. 4h). This result indicated that overexpression of CDC42-DN form might suppress the expression of endogenous CDC42 in ovaries. Meanwhile, the binding efficiency of CDC42 and p110β was markedly increased in CDC42-CA ovaries, confirmed our finding that CDC42 regulates the activity of PI3K signaling by binding to p110β with its active form (Fig. 4h). Taking together, these results revealed that the active form of CDC42 binds and activates p110β activity to control the activation of primordial follicles in mouse ovaries.

### CDC42 regulates PTEN expression level in oocytes of primordial follicles

**Fig. 5:**
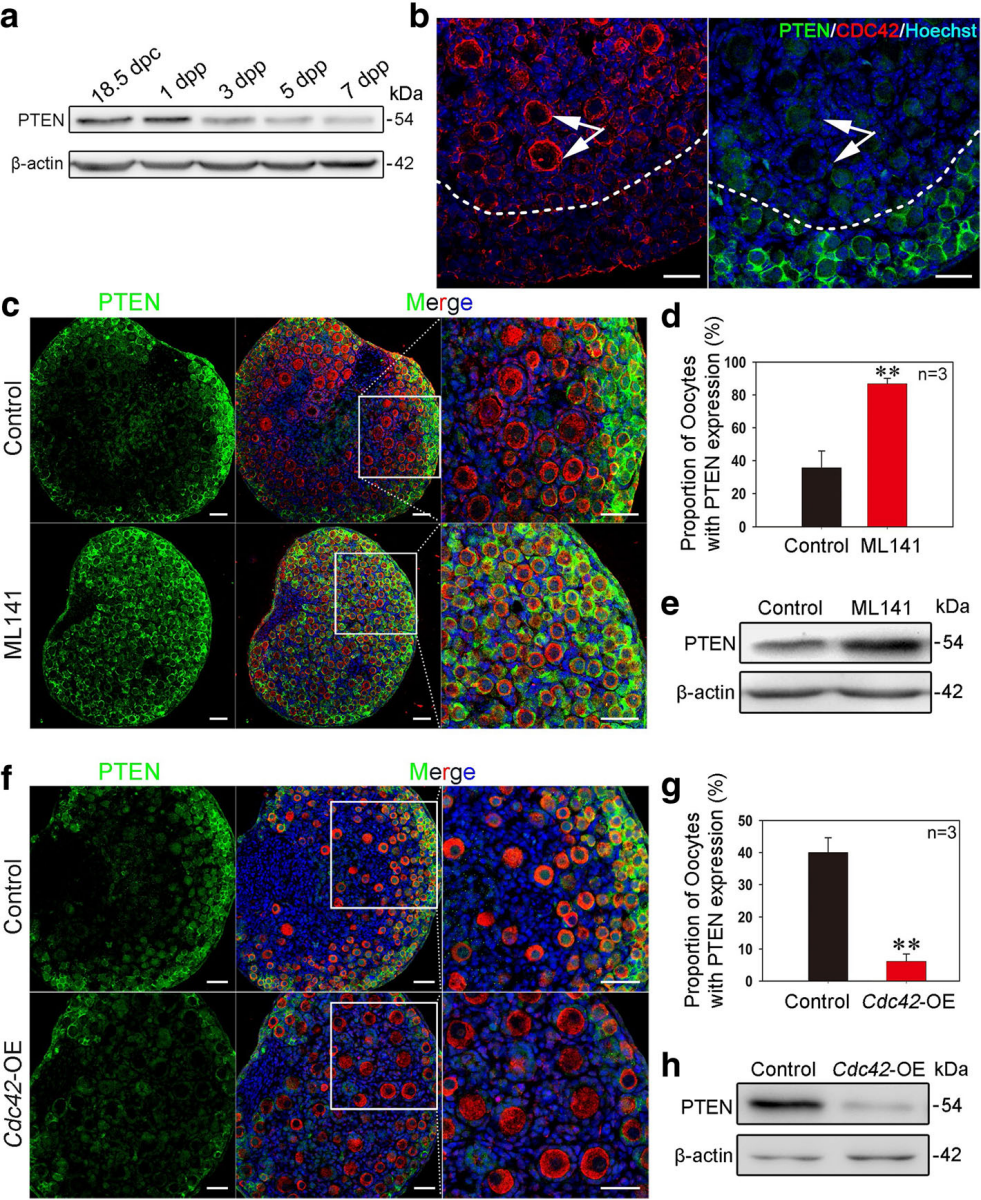
CDC42 regulates PTEN expression levels in oocytes of primordial follicles. **a** The expression of PTEN gradually decreased with the development of postnatal ovaries in mice. **b** CDC42 and PTEN were expressed in a mutually exclusive manner in oocytes of follicles. Ovaries at 3 dpp were sectioned and stained for PTEN (green) or CDC42 (red) in adjacent sections. Nuclei were dyed with a Hoechst counter-stain (blue). High expression of CDC42 was observed in activated oocytes (arrows) in the medulla region, whereas PTEN was mainly expressed in dormant oocytes in the cortical region of ovaries. **c** Suppression of CDC42 increased the expression of PTEN in ovaries. The ovaries were collected at 2 dpp and then cultured with or without ML141 for 5 days. Almost all oocytes exhibited a high expression of PTEN (green) in the ML141 group. **d** Oocyte counting results showed that the proportion of PTEN-positive oocytes increased from 36.08 ± 7.26% in the control group to 86.90 ± 3.32% in the ML141 group (Additional file 10: individual data values). **e** Western blot showed that PTEN expression was increased in 7 dpp ovaries after 5 days of ML141 treatment compared with controls. **f** Over-expressing CDC42 decreased the expression of PTEN in ovaries. One dpp ovaries were transfected with empty lentivirus or a lentiviral construct expressing *Cdc42* (*Cdc42*-OE) for 2 days or 6 days in vitro. Few oocytes with PTEN expression were observed in *Cdc42*-OE ovaries. **g** The proportion of oocytes with PTEN expression was significantly decreased in *Cdc42*-OE ovaries (6.61 ± 2.30%) compared with the control (40.28 ± 4.60%) (Additional file 10: individual data values). **h** PTEN expression was downregulated in *Cdc42*-OE-treated ovaries compared to controls. The experiments were repeated at least three times, and representative images are shown. Scale bars, 50 μm

As a major negative regulator of PI3K signaling, PTEN functions as a dominant suppressor in the process of primordial follicle activation. Ablation of PTEN from oocytes causes premature activation and depletion of primordial follicles in mice [5]. Given that CDC42 controls the activation of primordial follicles through PI3K signaling, we next examined whether CDC42 plays a role in regulating PTEN expression in oocytes. We first measured the localization of PTEN in neonatal ovaries. Immunostaining results showed that PTEN localized to the intracellular membrane of dormant oocytes in primordial follicles (Additional file 4: Figure S4c, arrowheads). No PTEN expression was observed in oocytes of activated follicles (Additional file 4: Figure S4c). Meanwhile, Western blot results revealed that the expression of PTEN decreased as follicle activation increased from 3 to 7 dpp in early ovarian development (Fig. 5a). These results confirmed previous reports that PTEN in oocytes suppressed the activation of primordial follicles in the ovary [5]. To investigate the relationship between CDC42 and PTEN, the expression patterns of both proteins were compared in 3 dpp ovaries under physiological conditions. Interestingly, a mutually exclusive expression of CDC42 and PTEN was observed in the oocytes of follicles. High-CDC42 expression was found in activated oocytes in the medulla region (Fig. 5b, left), whereas PTEN was mainly expressed in dormant oocytes of primordial follicles in the cortical region of ovaries (Fig. 5b, right). These results indicate that increased expression of CDC42 might regulate the expression of PTEN in oocytes to control the activation of primordial follicles.

To test our hypothesis, we next detected PTEN expression in ML141-treated and *Cdc42*-OE ovaries by immunofluorescence staining. We found that PTEN was expressed in almost all oocytes after 5 days of ML141 treatment (86.90 ± 3.32%), beginning at 2 dpp, compared to controls (36.08 ± 7.26%) (Fig. 5c, d). Western blot analysis showed that PTEN expression was remarkably increased in ML141-treated ovaries compared to controls (Fig. 5e). In contrast, the proportion of PTEN-positive oocytes was significantly decreased in *Cdc42*-OE ovaries after culture (Fig. 5f, g, 6.61 ± 2.30% vs. 40.28 ± 4.60%). PTEN protein expression was also decreased in *Cdc42*-OE ovaries compared with controls (Fig. 5h). These results indicate that CDC42 stimulates the activation of primordial follicles through regulation of PTEN expression levels to control PI3K signaling activity in oocytes.

### Short-term treatment with a CDC42 activator improves the activation of primordial follicles in neonatal and adult ovaries

**Fig. 6:**
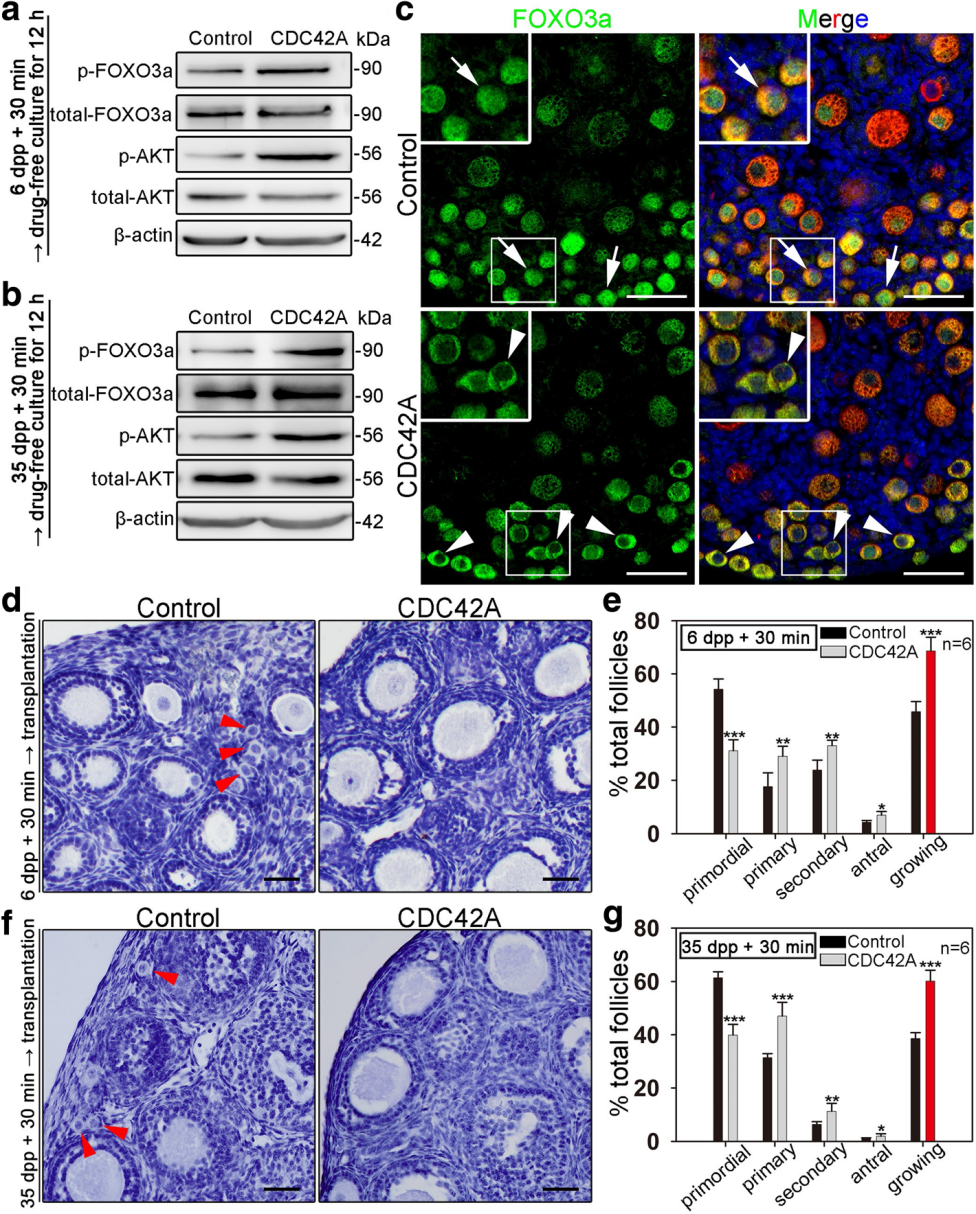
In vitro activation of primordial follicles in neonatal or adult ovaries by short-term treatment (30 min) with CDC42 activator. The mouse ovaries were collected at 6 or 35 dpp (age of adult). Six dpp intact ovaries or 35 dpp ovarian pieces were cultured in vitro. **a**, **b** After 30 min of CDC42 activator treatment followed by 12 h of drug-free culture in vitro, the phosphorylation of FOXO3a and AKT in ovaries was significantly increased in both 6 dpp ovaries (**a**) and 35 dpp ovaries (**b**). **c** After CDC42 activator treatment, FOXO3a translocated to the cytoplasm in most oocytes in the ovarian cortex of 6 dpp ovaries (arrowheads) compared with control groups (arrows). **d**, **f** After 30 min of CDC42 activator treatment, intact ovaries at 6 dpp or fragmented ovaries at 35 dpp were immediately transplanted under the kidney capsules of ovariectomized adult hosts. After 2 weeks of in vivo development, the cluster of primordial follicles was hardly observed in cortex of both 6 dpp (**d**) and 35 dpp (**f**) ovaries after CDC42 activator treatment. **e**, **g** Follicle counting results showed the distribution of follicles after treatment with or without CDC42 activator in ovaries. The proportion of primary, secondary, and antral follicles was significantly increased in activator-treated ovaries compared to control groups (Additional file 10: individual data values). The experiments were repeated at least three times, and representative images are shown. Scale bars, 50 μm

Previous results showed that primordial follicles in ovaries could be activated by PI3K or mTOR stimulators in vitro [11, 28]. Since our results demonstrated that CDC42 plays a key role in controlling the activation of primordial follicles, we next tested whether a CDC42 activator could increase primordial follicle activation in vitro. The Rac/Cdc42 activator II (Cdc42 activator), an epidermal growth factor (EGF)-mediated CDC42 activator, was used to treat mouse ovaries. In contrast with previous reports of in vitro activating stimulators which require long-term treatment (PI3K activator bpV (pic) or mTOR activators PA and PRO for 24-h treatment) [10, 28], CDC42 activator has been reported as a rapid stimulator in various cell lines [29, 30]. Therefore, we first investigated whether a short-term treatment with the drug could stimulate the activation of oocytes at the molecular level. Intact 6 dpp ovaries or fragmented 35 dpp ovaries were incubated with the activator for 30 min in vitro, followed by culture in drug-free medium for 12 h. A dramatic increase in p-AKT (Fig. 6a, b) and a clear shuttling of FOXO3a from the nucleus to the cytoplasm (Fig. 6c, arrowheads) were observed in treated ovaries, indicating that a short-term treatment with CDC42 activator is able to stimulate the activation of primordial follicles.

To further investigate the developmental potential of follicles after CDC42 activator stimulation, the treated ovaries were allo-transplanted under the kidney capsules of adult bilaterally ovariectomized mice. Two weeks after transplantation, an identical number of total follicles was found in activator-treated 6 dpp ovaries and control ovaries, indicating that the drug has no effect on follicle survival (Additional file 6: Figure S6). Histological analyses showed that primordial follicles were hardly observed in the cortex of CDC42 activator-treated intact 6 dpp (Fig. 6d) or fragmented 35 dpp ovaries (Fig. 6f), which is in sharp contrast to the many primordial follicles found in control ovaries (Fig. 6d, f, arrows). Follicle counting results from grafted mice also showed major increases in the percentage of growing follicles including primary, secondary, and antral stages in activator-treated ovaries compared to control groups (Fig. 6e, g and Additional file 7: Table S1). These results indicated that CDC42 activator could be used as a short-term stimulator to activate primordial follicles in vitro.

## Discussion

As the most abundant fertility reserve in female mammals, dormant primordial follicles can be found in the ovaries of both aged women and POI patients, making them an ideal source for clinical treatment of female infertility [31–35]. To utilize the dormant primordial follicles, the follicles must be activated for further development [10]. In this study, we have demonstrated that oocyte-expressed CDC42 plays a functional role in regulating primordial follicle activation by manipulating the activity of PI3K signaling in dormant oocytes. We also showed that CDC42 might be a highly efficient target to be used in IVA approach since short-term treatment with CDC42 activator increased the activation of primordial follicles in mice. CDC42, a well-studied small GTPase, has been reported to play a role in multi-cellular events such as cytoskeletal changes, membrane trafficking, and mRNA transcription. With regards to female germ cell development, a previous study reported that deletion of CDC42 in activated oocytes via follicle-specific *Zp3-Cre* disrupts oocyte maturation and leads to complete female infertility [21]. However, loss of CDC42 in growing oocytes has no effect in late folliculogenesis since a normal follicle distribution is observed in *Zp3-Cre;Cdc42*^*fl/fl*^ ovaries [21]. Our data from the current study reveal that CDC42 also plays a functional role in initiating the activation of oocytes. Therefore, it seems that CDC42 plays a temporal role in ovarian folliculogenesis for both oocyte activation and maturation.

In the last decade, the mechanisms of primordial follicle activation have been revealed at both the cellular and molecular levels, and a two-cell–two-pathway model has been proposed as controlling the process [6]. In the first step, the levels of mTORC1 signaling activity in preGCs decide whether or not a primordial follicle will be recruited into the growing follicle pool [4]. The second step involves the awakening of dormant oocytes by the upregulation of oocyte PI3K signaling through KITL-KIT [4, 36]. In the current study, we found that CDC42 regulates the activation of primordial follicles by awakening dormant oocytes via stimulation of PI3K signaling in primordial follicles. In COS7 cells, CDC42 could bind to phosphoinositide 3-kinase [37]. Further investigation showed that CDC42 could bind to p110β in a GTP-dependent manner, which directly increased PI3K activity in mouse embryonic fibroblasts [25]*.* Our results showed that p110β localized to the intracellular membrane in the oocytes of activated follicles and that CDC42 could bind to the PI3K catalytic subunit p110β to activate PI3K pathway in vivo. Thus, we concluded that activated CDC42 is functionally capable of recruiting p110β to the cell membrane, leading to an increase in PI3K signaling activity. In addition to binding to p110β directly, we also found that CDC42 participates in downregulating PTEN expression in oocytes of dormant primordial follicles. Although it is unknown whether CDC42 directly regulates the expression of PTEN, it has been reported that PTEN was excluded from the leading edge of mouse chemotaxing neutrophils by CDC42 [16]. This is consistent with our finding that CDC42 and PTEN expression is mutually exclusive in oocytes of follicles. Co-IP assay shows that CDC42 does not bind to PTEN directly in ovaries (data not shown), but this does not rule out the possibility that CDC42 regulates PTEN phosphatase enzyme activity through other molecular interaction.

Under physiological conditions, the activation of primordial follicles is initiated by the upregulation of mTORC1 signaling in preGCs [4]. However, it seems that stimulation of related pathways in either oocytes or somatic preGCs is able to activate primordial follicles in vitro since both the activators of PI3K and mTORC1 signaling are used in IVA treatment [28]. Meanwhile, the activating efficiencies of PI3K and mTORC1 activators are similar in previous reports, which show that the ovarian tissue pieces need to be treated for more than 24 h in vitro. In contrast to the previous IVA activators, our data showed that the CDC42 activator could significantly improve the activation rate of primordial follicles in mice with a short-term treatment, which is consistent with previous reports that the CDC42 activator is a rapid stimulator in various cell lines [29, 30]. Therefore, we propose that CDC42 is a potent target for a highly efficient and short-term treatment approach to IVA in clinical practice. Although our results revealed that CDC42 stimulates the activation of oocytes by regulating PI3K signaling, the mechanisms of how short-term CDC42 activator treatment increases the activity of PI3K signaling in ovaries is not completely clear. The CDC42 activator used in our study, Rac/Cdc42 activator II, is an epidermal EGF-mediated CDC42 activator. It is possible that the activator directly stimulates the receptor, such as EGF receptor on the cell surface, and causes a cascading reaction to ensure quick activation of PI3K. Meanwhile, we found that CDC42 expression is mainly localized but not limited in oocytes in ovaries. CDC42 also expresses in granulosa cells in growing follicles and might also function in granulosa cells during folliculogenesis. As the whole ovarian tissue was treated with CDC42 activator, the exact mechanisms of IVA approach we applied in the study might be a combination effect of both oocytes and granulosa cells. Additionally, to verify the effect of CDC42 activator in adult ovaries, we fragmented 35 dpp ovaries for culture in our study. A previous study reported that fragmentation of murine ovaries could disrupt ovarian Hippo signaling, leading to increased expression of downstream growth factors, promotion of follicle growth [12]. Fragmentation and treatment of CDC42 activator may play a combining role in stimulating primordial follicle activation in our study.

## Conclusions

In the current study, we established a system to study the activation of mammalian primordial follicles by culturing the whole ovaries from neonatal mice. Using this system, our results have revealed that CDC42 in oocytes plays a regulatory role in the activation of primordial follicles and controls the development of dormant oocytes in mice. This is achieved by binding PI3K catalytic subunit p110β to improve the activity of PI3K signaling in oocytes. Meanwhile, our study suggests that the CDC42 activator is a potent short-term stimulator for the activation of primordial follicles in vitro to treat infertility in POI patients.

## Methods

### Animals

All CD1 mice were purchased from the Laboratory Animal Center of the Institute of Genetics in Beijing and kept in mouse facilities that met the requirements of the China Agricultural University Institutional Animal Care and Use Committee. Mice were housed in China Agricultural University with 16/8-h light/dark cycles, at 26 °C, with free access to food and water (Rat & Mouse Maintenance Diet 1022, HFK Bio-tech, China). Female mice (6 to 8 weeks old) were mated with adult males at a ratio of 1:1 overnight. Mice with a vaginal plug in the next morning were defined at 0.5 day post-coitus (dpc). The day after partum was considered to be 1 day post partum (dpp).

### Ovary culture

Neonatal females were sacrificed by cervical dislocation on the designated time. Mouse ovaries were separated by microdissection in cold phosphate buffered saline (PBS) under a stereomicroscope (ZSA302, COIC, China) in sterile conditions. The isolated ovaries were cultured on an insert (PICM0RG50, Millipore, USA) in 6-well culture dishes (NEST, China) in 1200 μL Dulbecco’s modified Eagle’s medium/Ham’s F12 nutrient mixture (DMEM/F12) (GIBCO, Life Technologies, USA) plus ITS (1:100, Sigma, USA) and Penicillin-Streptomycin Solution at 37 °C, 5% CO2 and saturated humidity. Ovaries were cultured for 2 or 5 days to assess the role of CDC42, in either medium alone or medium supplemented with ML141 (5uM, Selleck, China) or ZCL278 (50uM, Selleck, China). ML141 is a selective reversible CDC42 inhibitor. ZCL278 is a selective CDC42 inhibitor.

### Histological staining and follicle counts

Ovaries were fixed in 4% PFA overnight, embedded in paraffin, and sectioned serially at 5 μm. After stained with hematoxylin, every fifth section was analyzed for the presence of oocytes and follicles. The counting results were multiplied by five to estimate the total numbers of oocytes and follicles in each ovary. The follicles were distinguished from each other as follows: primordial follicle (a single oocyte surrounded by several flattened pre-granulosa cells) and activated follicle (an enlarged oocyte surrounded by a mixture of squamous and cuboidal somatic cells or an enlarged oocyte surrounded by one or several layers of cuboidal granulosa cells).

Ovarian follicles at different stages of development, including primordial (type 2), primary (type 3), secondary (type 4 and 5), and antral (type 6 and 7) follicles were counted in all sections of an ovary based on the well-accepted standards established by Pedersen and Peters [38].

### Immunofluorescence staining

Fresh separated ovaries were fixed in cold 4% PFA overnight, embedded in paraffin, and serially sectioned at 5 μm. The sections were deparaffinized, rehydrated, and subjected to high temperature (95–98 °C) antigen retrieval with 0.01% sodium citrate buffer (pH 6.0). The sections were then blocked with ADB [3% BSA, 1% normal donkey serum in TBS (0.05 M Tris-HCl pH 7.6 and 0.15 M NaCl)] for 60 min at room temperature and incubated with primary antibodies for 12–16 h at 4 °C. The antibodies were diluted as follows: anti-CDC42 antibody (Abcam, UK) at 1:250, DDX4 (mouse, 1:200, Abcam, UK), anti-BrdU antibody (Abcam, UK) at 1:200, and anti-p-FOXO3a antibody (Cell Signaling Technologies, USA) at 1:400, anti-PTEN antibody (Cell Signaling Technologies, USA) at 1:200, anti-p110β antibody (Abcam, UK) at 1:200. Subsequently, after rinsing thoroughly with PBS, the sections were incubated with fluorophore-conjugated secondary antibody dissolved in ADB for 2 h at 37 °C. The antibodies were diluted as follows: Alexa Fluor 488- or 555-conjugated donkey secondary antibodies against mouse and rabbit IgG (1:200, Life Technologies, USA). Slides were then rinsed in PBS, stained with Hoechst 33342 (B2261, Sigma, USA) for 5 min and sealed in anti-fade fluorescence mounting medium (Applygen, China) with coverslips. Sections were examined and photographed using Nikon Eclipse 80i digital fluorescence microscope or Nikon A1 laser scanning confocal microscope. Antibodies were listed in Additional file 8: Table S3.

### qRT-PCR

Eight ovaries in each group were extracted by TRIZOL Reagent (Invitrogen, Life Technologies, USA) according to the manufacturer’s protocol. cDNA was reverse transcripted using 1 μg total RNA (Promega Reverse Transcription System, Promega, USA). Quantitative RT-PCR reaction were operated and analyzed by Applied Biosystems 7300 Real-Time PCR System (Life Technologies, USA). Data were normalized by *β-actin*. Primers were listed in Additional file 9: Table S2.

### RNAi knockdown of *Cdc42* in ovaries

Ovaries at 1 dpp were injected with 0.3 μl *Cdc42* esiRNAs (200 ng/μL, diluted in PBS), a cocktail of siRNAs (Sigma, USA), or scrambled siRNA by a thin glass needle with a mouthpiece. After this, ovaries were performed electric transfection (ECM2001, BTX, CA) to assist siRNA transfection. The parameters of the electric transfection were three 5-ms-long quasi-square pulses at a pulse-field strength of up to 40 V/cm. The efficiency of RNAi was detected by qRT PCR after 24-h culture and Western blot after 48-h culture.

### Lentiviral production and ovary infection

Lentiviruses were produced in 293 T cells by co-transfecting 5 μg pMD2.G, 15 μg psPAX2, and 20 μg of the transfer vector (pSicoR or pLVX-IRES-ZsGreen1). CDC42 overexpressing lentiviruses were constructed by cloning open reading frame of *Cdc42* into the pLVX-IRES-ZsGreen1 vector. This lentivirus expressing *Cdc42* followed a GFP tag. Therefore, cells expressing CDC42 were also labeled with GFP. The transfection was performed by Lipofectamine 3000 (Invitrogen, USA), and the transfection media were replaced 6 h post transfection. The viral supernatants were harvested at 24 and 48 h with 0.45-μm membrane filtration and centrifuged at 40,000 rpm at 4 °C for 2 h [39]. The lentiviruses were injected into the ovary using a thin glass needle with a mouthpiece. For each injection, the optimal volume was 0.3 μL per ovary. The lentiviral construct pMD2.G and psPAX2 were obtained from Dr. Sheng Cui (China Agricultural University). The lentiviral construct pSicoR and pLVX-IREX-ZsGreen1 were obtained from Dr. Haibin Wang (Xiamen University).

### Immunoblotting and CDC42 activity detection

Mouse ovaries were homogenized in WIP Tissue and Cell lysis solution containing 1 mM phenylmethylsulfonyl fluoride (Cell Signaling Technologies, USA). Next, proteins were prepared according to the manufacturer’s instructions. The concentration of proteins was measured by a BCA assay (Beyotime, China). Samples containing 50 μg protein were separated by 10% SDS-PAGE and transferred to polyvinylidene fluoride (PVDF) membranes (IPVH00010, Millipore, USA). The membranes were then incubated overnight at 4 °C with the appropriate primary antibody listed below: CDC42 (21 kDa, 1:300, Abcam, UK), p-AKT (56 kDa, Thr308, 1:500, Beyotime, China), AKT (56 kDa, 1:500, Beyotime, China), p-FOXO3a (90 kDa, 1:300, Santa Cruz Biotechnology, USA), FOXO3a (90 kDa, 1:200, Santa Cruz Biotechnology, USA), PTEN (54 kDa, 1:200, Cell Signaling Technologies, USA), and p110β (123 kDa, 1:200, Abcam, UK). After rinsing thoroughly with TBST, the membranes were incubated with secondary antibody (1:5000, ZSGB-BIO, China). Membranes were visualized using SuperSignal West Pico chemiluminescent detection system (Prod 34080, Thermo, USA). β-actin (42 kDa, Sigma, USA) was used as an intrinsic control. An Alpha Imager 2200 was used to quantify the relative amount of protein. Antibodies were listed in Additional file 8: Table S3.

CDC42 activity (CDC42-GTP) was determined using RAC1/CDC42 Activation Magnetic Beads Pull-down Assay Kits from Merck Millipore following the instructions. In brief, 100 μg of clarified total ovary lysates were incubated with PAK-1 PBD magnetic beads in microfuge tube at 4 °C for 45 min in a spin column. The beads were pelleted by setting the tubes on a magnetic tube stand for 10 s. Removed and discarded the supernatant. The magnetic beads were washed for three times with MLB buffer. The magnetic beads were resuspended in 40 μL of 2X Laemmli reducing sample buffer and boil for 5 min. Addition of 2 μL of 1 M dithiothreitol prior to boiling. The samples were run on a 10% SDS-PAGE separating gel and subjected to Western blot analyses using an antibody against CDC42. CDC42 activity was indicated by the amount of PAK1-PBD-bound CDC42 (GTP-CDC42). Total ovary lysates were also directly immunoblotted for total CDC42 levels for normalization.

### Immunoprecipitation

Magnet beads (Life Technologies, USA) were washed and resuspended in 200 μL Ab Binding and Washing Buffer containing 5 μg Ab. The mix were incubated 30 min with rotation at RT. Supernatant were removed and magnet beads-Ab complex were washed by 200 μL Ab Binding and Washing Buffer. The same amount of ovaries (35 each group or 70 total) were lysed in 200 μL IP buffer, and lysates were cleared by centrifugation, 10 μL of the lysates were kept as input. Supernatant were removed from magnet beads, and ovary lysates were added to beads-Ab complex, gently resuspended by pipetting and incubated 30 min at RT with rotation. Supernatant were transferred to a clean tube, and beads-Ab-Ag complex was washed three times, using 200 μL washing buffer. The washing buffer was removed, and the beads-Ab-Ag complex was gently resuspended in 20 μL elution buffer. Incubate 5 min at RT. 5x SDS sample buffer were added to the mix and the tubes were placed at 100 °C for 10 min. Then, the samples were separated by SDS-PAGE and detected by relevant antibodies (Additional file 8: Table S3).

### In vitro transient treatment of mouse ovaries and ovarian kidney capsules transplantation

Paired ovaries from mice at 6 dpp were collected, and one ovary served as control and the other was incubated with CDC42 activator II (1 IU, Cytoskeleton, USA) for 30 min on an insert (PICM0RG50, Millipore, USA) in six-well culture dishes. Ovaries from mice at 35 dpp were cut into 4 pieces before treatment. The culture media was DMEM/F12 (GIBCO, Life Technologies, USA) supplemented with ITS (1:100, Sigma, USA) and penicillin-streptomycin solution. After the treatment, paired ovaries from the same donor were randomly inserted under each kidney capsule of the same ovariectomized host. Mice were sacrificed at 14 days after transplantation to assess follicular development. For Western blot assay, ovaries were incubated with or without CDC42 activator II on an insert in six-well culture dishes in culture media described above for 30 min, respectively. Ovaries were transferred to drug-free medium and cultured for 12 h and then harvested to Western blot analyses.

### Statistical analysis

All experiments were repeated at least three times, and the values are presented as the means ± SEM. Data were analyzed by *t* test and considered statistically significant at *P* < 0.05.

## Additional files

Additional file 1: **Figure S1.** (a): Histological and follicle counting analysis showed comparable dynamics of follicle development in in vitro culture compared with in vivo. Immunofluorescent staining of ovaries at 2 and 7 dpp in vivo, and ovaries that were collected at 2 dpp followed 5 days in vitro culture. Oocytes were stained with DDX4 (red), and nuclei were dyed with a Hoechst counter-stain (blue). Normal follicle distribution was found in both 7 dpp ovaries and cultured ovaries. b and c The total oocytes and activated follicles were quantified. Quantification results showed a comparable number of both total oocytes and activated follicles in ovaries developed in vivo or in vitro (Additional file 10: Individual data values). The experiments were repeated at least three times, and representative images are shown. Scale bars, 50 μm. (PDF 852 kb)

Additional file 2: **Figure S2.** The sections of ovaries in different groups were stained by hematoxylin to detect the morphology. (a) Ovaries at 2 dpp were cultured in media alone (control) or with CDC42 inhibitor ML141 or ZCL278 for 5 days in vitro. The activation of oocytes was remarkably suppressed in ML141 or ZCL278-treated ovaries, and few activated oocytes were observed in these ovaries compared to the control. (b) Ovaries at 1 dpp were injected with *Cdc42* esiRNA (*Cdc42-*KD) and cultured for 6 days. Histological analysis showed that few activated follicles were observed in the *Cdc42-*KD ovaries compared with the control. (c) Ovaries at 1 dpp were transfected with control empty lentivirus or a lentiviral construct expressing *Cdc42* (*Cdc42-*OE) for 6 days in vitro. Morphological analysis showed that ovaries in the *Cdc42*-OE group exhibited more activated follicles than the control. The experiments were repeated at least three times, and representative images are shown. Scale bars, 50 μm. (PDF 1389 kb)

Additional file 3: **Figure S3.** Suppressing CDC42 activity by ML141 treatment decreased the PI3K signaling activity in fragmented 35 dpp ovaries. (a) Western blot showed that the phosphorylation of FOXO3a and AKT were decreased in ML141-treated ovaries. (b) Nuclear localization of FOXO3a (white arrows) was observed in oocytes of ML141-treated fragmented ovaries indicating suppressed PI3K signaling activity in oocytes of primordial follicles. Oocytes were stained with DDX4 (red). Nuclei were dyed with a Hoechst counter-stain (blue). The experiments were repeated at least three times, and representative images are shown. Scale bars, 50 μm. (PDF 603 kb)

Additional file 4: **Figure S4.** (a) Ovaries at 5 dpp were stained for p110β (green) and the oocyte marker DDX4 (red). Nuclei were dyed by a Hoechst counter-stain (blue). The expression of p110β distributed in cytoplasm of dormant oocytes in primordial follicles (arrowheads) and shuttled to intracellular membrane of oocytes in activated follicles (arrows). (b) Suppressing CDC42 activity had no effect on the expression of p110β. (c) Immunostaining showed that PTEN was mainly expressed in the dormant oocytes of primordial follicles. Ovaries were stained for PTEN (green) and the oocyte marker DDX4 (red) at the indicated time points. Nuclei were dyed by a Hoechst counter-stain (blue). PTEN concentrated upon the intracellular membrane of oocytes in primordial follicles (arrowheads). The experiments were repeated at least three times, and representative images are shown. Scale bars, 40 μm. (PDF 1146 kb)

Additional file 5: **Figure S5.** Transfection of CDC42-DN (CDC42T17N) or CDC42-CA (CDC42Q61L) lentivirus constructs disrupts the activation of primordial follicle through regulating PI3K signaling in neonatal ovaries. (a) The ovaries at 1 dpp were transfected with empty lentivirus, lentiviral construct expressing CDC42-DN or CDC42-CA for 2 days in vitro. Green fluorescence from the GFP reporter was observed in ovaries following 2 days of lentiviral infection indicating a satisfactory efficiency of lentivirus transfection. (b) Oocyte counting results showed a comparable number of total oocytes in control, CDC42-DN, and CDC42-CA ovaries after 6 days of treatment. (c and d) Transfection of CDC42-DN and CDC42-CA affected the activity of PI3K signaling in ovaries. CDC42-DN weakened the nucleus-cytoplasm shuttle of FOXO3a (green fluorescence) in the oocytes, whereas CDC42-CA increased the cytoplasmic localization of FOXO3a in transfected ovaries. Oocytes were stained with DDX4 (red). Nuclei were dyed with a Hoechst counter-stain (blue) (c). Immunoblotting results showed that the phosphorylation of FOXO3a and AKT was decreased in CDC42-DN ovaries but increased in CDC42-CA ovaries. Levels of total FOXO3a, AKT, and β-actin were used as internal controls (d). The experiments were repeated at least three times, and representative images are shown. Scale bars, 50 μm. (PDF 842 kb)

Additional file 6: **Figure S6.** The survival and development of ovarian follicles were not affected by CDC42 activator treatment. Ovaries were collected at 6 dpp and cultured with or without CDC42 activator for 30 min then transplanted under kidney capsules of ovariectomized adult hosts. After 2 weeks of transplantation, the number of total oocytes in ovaries was quantified. CDC42 activator-treated ovaries showed a comparable number of total oocytes compared to the control (Additional file 10: Individual data values). (PDF 464 kb)

Additional file 7: **Table S1.** Follicle counting results. (DOCX 15 kb)

Additional file 8: **Table S2.** List of primers used in qRT-PCR. (DOCX 14 kb)

Additional file 9: **Table S3.** List of antibodies. (DOCX 14 kb)

Additional file 10: Individual data values. (XLSX 30 kb)
