# Supplementary figures and images for: CDC42 controls the activation of primordial follicles by regulating PI3K signaling in mouse oocytes
Source: BMC Biol. 2018 Jul 5;16:73. doi: 10.1186/s12915-018-0541-4 (PMC6033292; doi:10.1186/s12915-018-0541-4)

**a**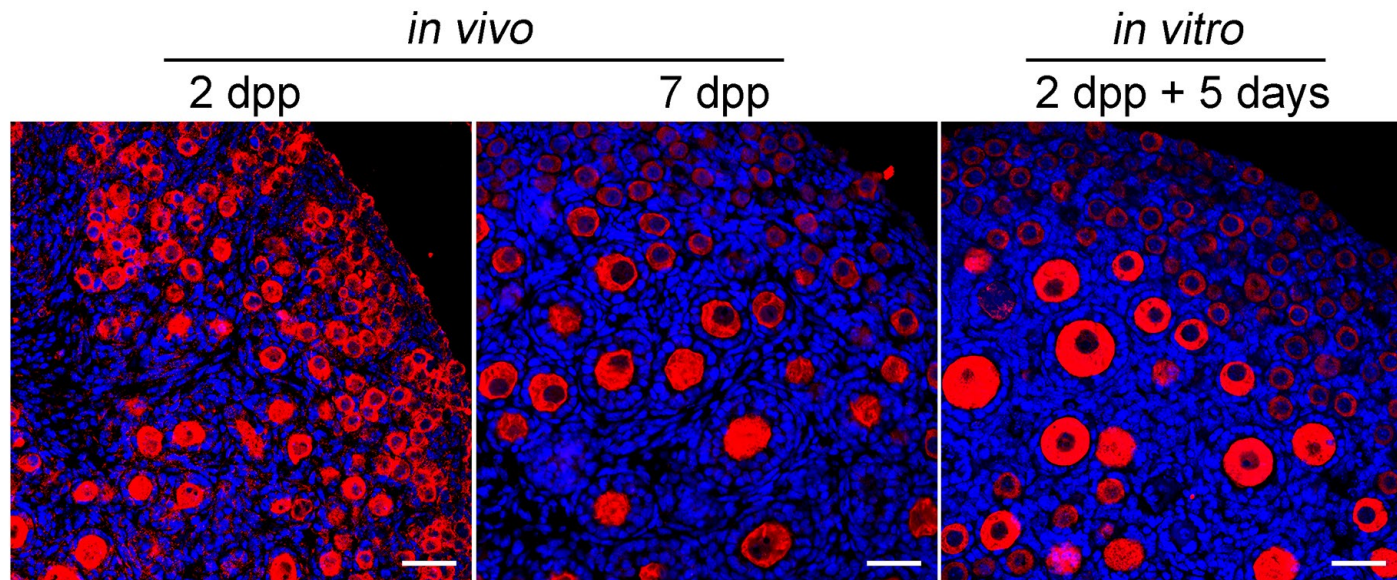**b**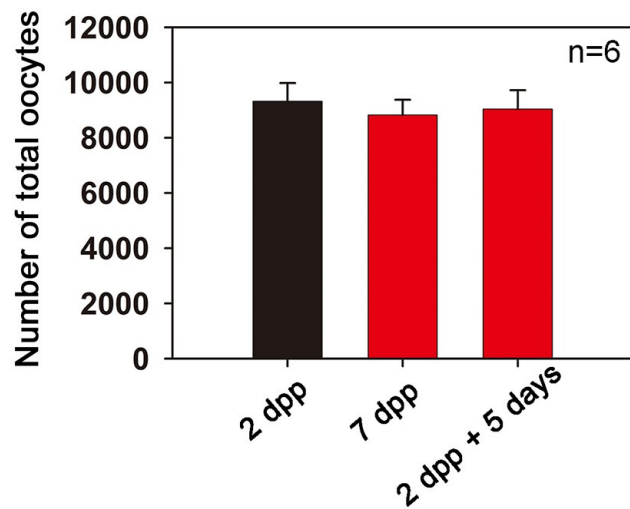**c**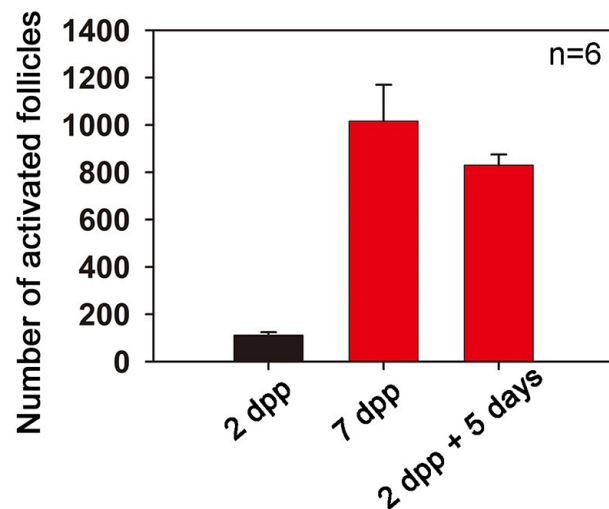

Supplement: Supplementary file 1 — Figure S1. (a): Histological and follicle counting analysis showed comparable dynamics of follicle development in in vitro culture compared with in vivo. Immunofluorescent staining of ovaries at 2 and 7 dpp in vivo, and ovaries that were collected at 2 dpp followed 5 days in vitro culture. Oocytes were stained with DDX4 (red), and nuclei were dyed with a Hoechst counter-stain (blue). Normal follicle distribution was found in both 7 dpp ovaries and cultured ovaries. b and c The total oocytes and activated follicles were quantified. Quantification results showed a comparable number of both total oocytes and activated follicles in ovaries developed in vivo or in vitro (Additional file 10: Individual data values). The experiments were repeated at least three times, and representative images are shown. Scale bars, 50 μm. (PDF 852 kb) [file 12915_2018_541_MOESM1_ESM.pdf]

**a**

Control

ML141

ZCL278

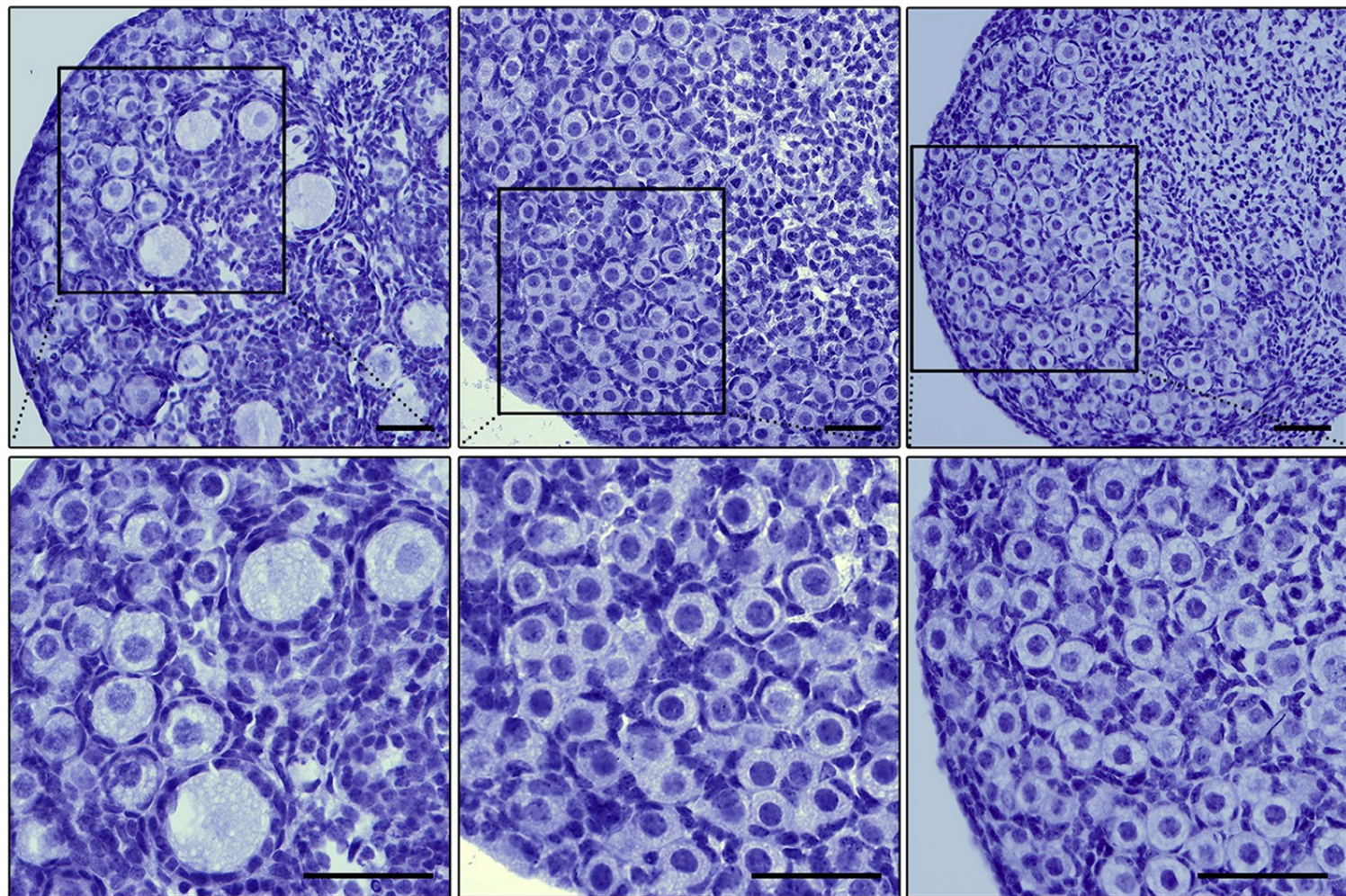**b**

Control

*Cdc42*-KD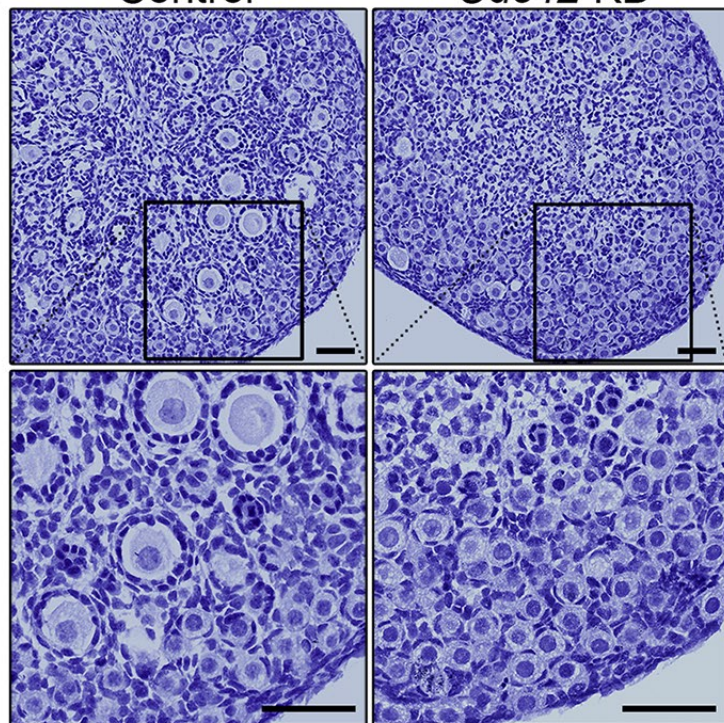**c**

Control

*Cdc42*-OE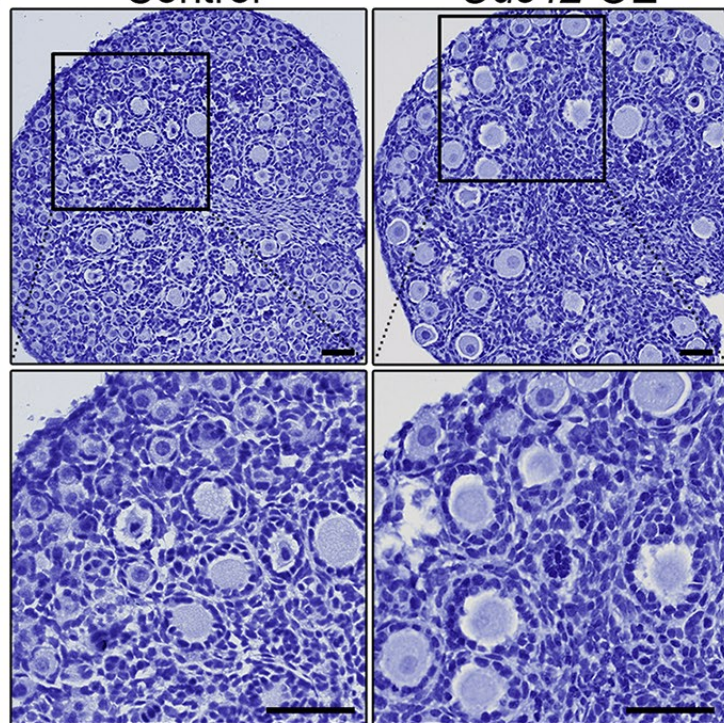

Supplement: Supplementary file 2 — Figure S2. The sections of ovaries in different groups were stained by hematoxylin to detect the morphology. (a) Ovaries at 2 dpp were cultured in media alone (control) or with CDC42 inhibitor ML141 or ZCL278 for 5 days in vitro. The activation of oocytes was remarkably suppressed in ML141 or ZCL278-treated ovaries, and few activated oocytes were observed in these ovaries compared to the control. (b) Ovaries at 1 dpp were injected with Cdc42 esiRNA (Cdc42-KD) and cultured for 6 days. Histological analysis showed that few activated follicles were observed in the Cdc42-KD ovaries compared with the control. (c) Ovaries at 1 dpp were transfected with control empty lentivirus or a lentiviral construct expressing Cdc42 (Cdc42-OE) for 6 days in vitro. Morphological analysis showed that ovaries in the Cdc42-OE group exhibited more activated follicles than the control. The experiments were repeated at least three times, and representative images are shown. Scale bars, 50 μm. (PDF 1389 kb) [file 12915_2018_541_MOESM2_ESM.pdf]

**a**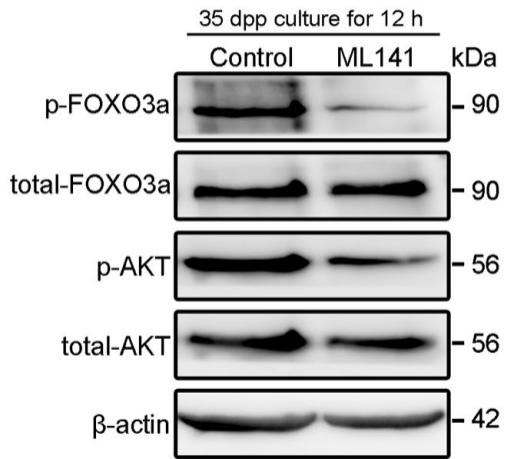**b**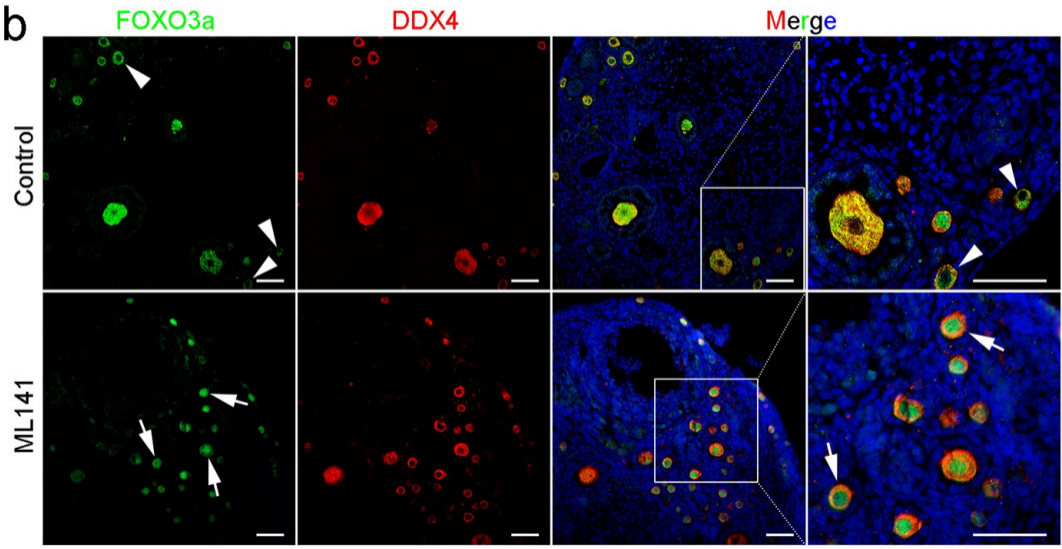

Supplement: Supplementary file 3 — Figure S3. Suppressing CDC42 activity by ML141 treatment decreased the PI3K signaling activity in fragmented 35 dpp ovaries. (a) Western blot showed that the phosphorylation of FOXO3a and AKT were decreased in ML141-treated ovaries. (b) Nuclear localization of FOXO3a (white arrows) was observed in oocytes of ML141-treated fragmented ovaries indicating suppressed PI3K signaling activity in oocytes of primordial follicles. Oocytes were stained with DDX4 (red). Nuclei were dyed with a Hoechst counter-stain (blue). The experiments were repeated at least three times, and representative images are shown. Scale bars, 50 μm. (PDF 603 kb) [file 12915_2018_541_MOESM3_ESM.pdf]

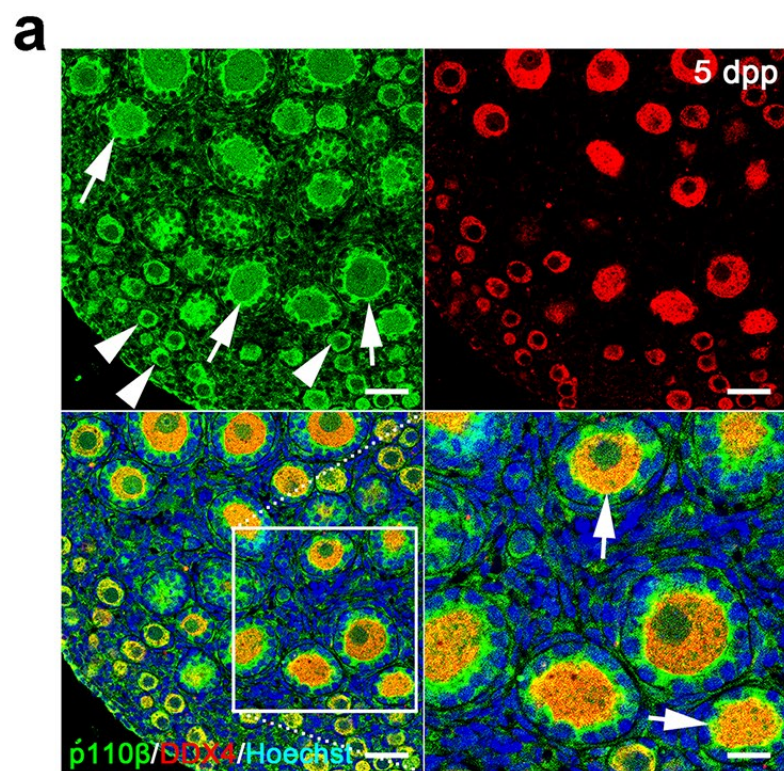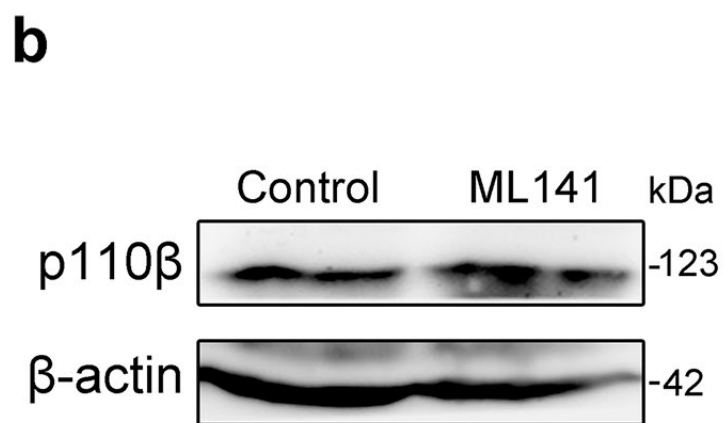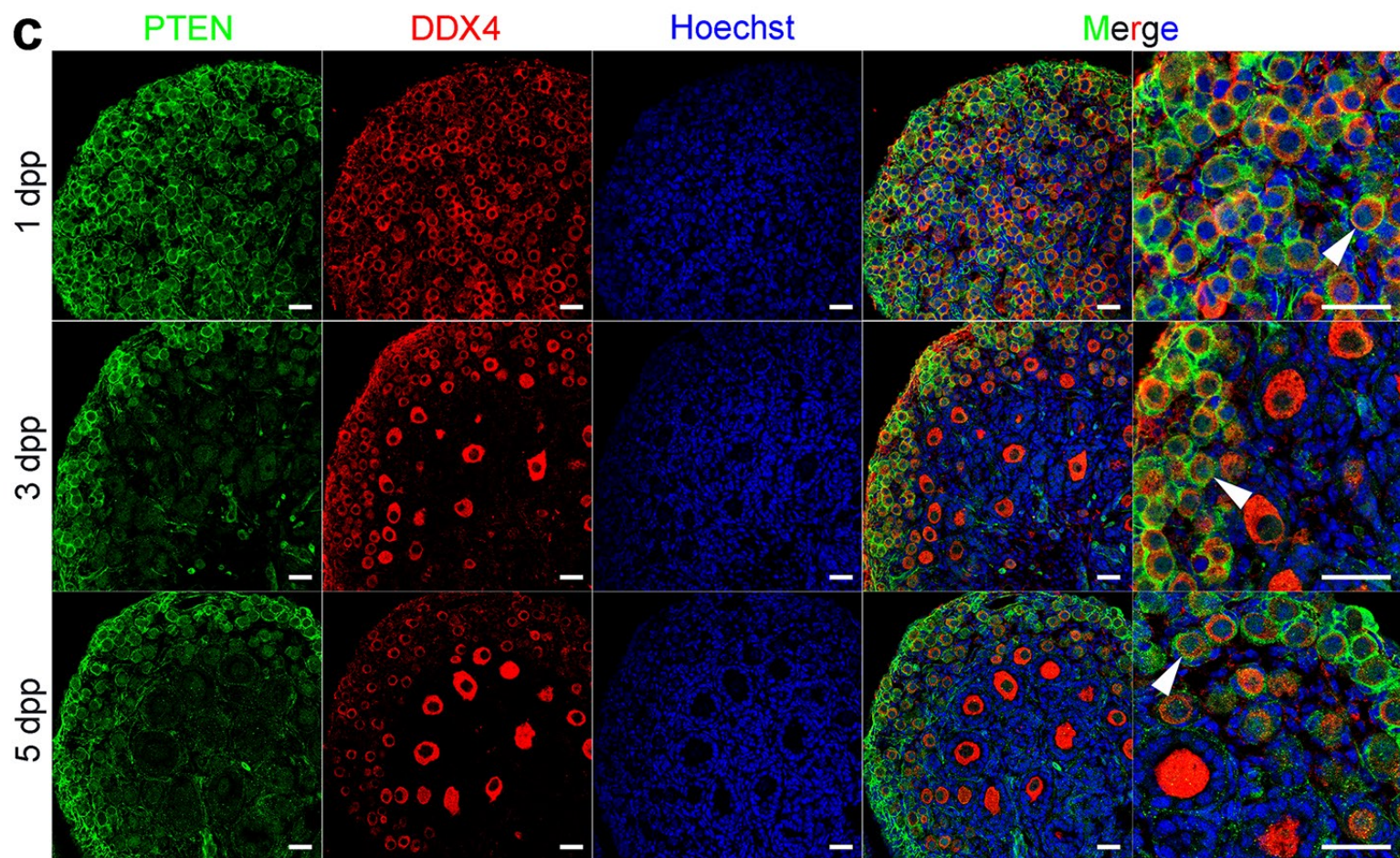

Supplement: Supplementary file 4 — Figure S4. (a) Ovaries at 5 dpp were stained for p110β (green) and the oocyte marker DDX4 (red). Nuclei were dyed by a Hoechst counter-stain (blue). The expression of p110β distributed in cytoplasm of dormant oocytes in primordial follicles (arrowheads) and shuttled to intracellular membrane of oocytes in activated follicles (arrows). (b) Suppressing CDC42 activity had no effect on the expression of p110β. (c) Immunostaining showed that PTEN was mainly expressed in the dormant oocytes of primordial follicles. Ovaries were stained for PTEN (green) and the oocyte marker DDX4 (red) at the indicated time points. Nuclei were dyed by a Hoechst counter-stain (blue). PTEN concentrated upon the intracellular membrane of oocytes in primordial follicles (arrowheads). The experiments were repeated at least three times, and representative images are shown. Scale bars, 40 μm. (PDF 1146 kb) [file 12915_2018_541_MOESM4_ESM.pdf]

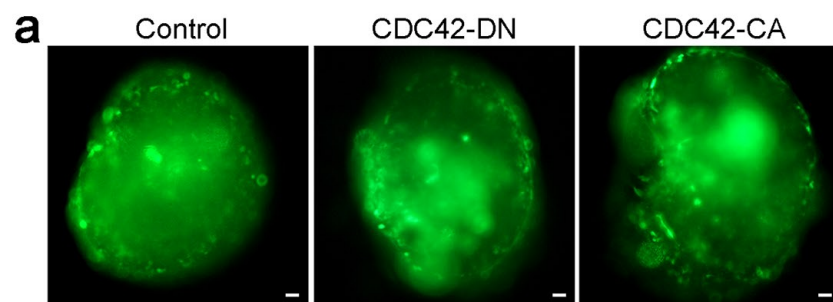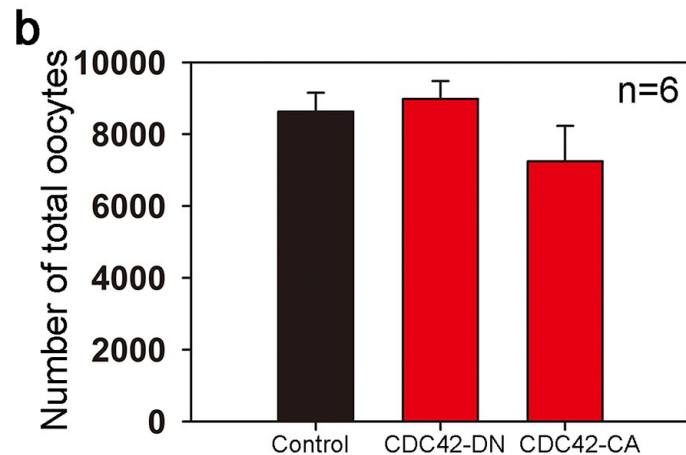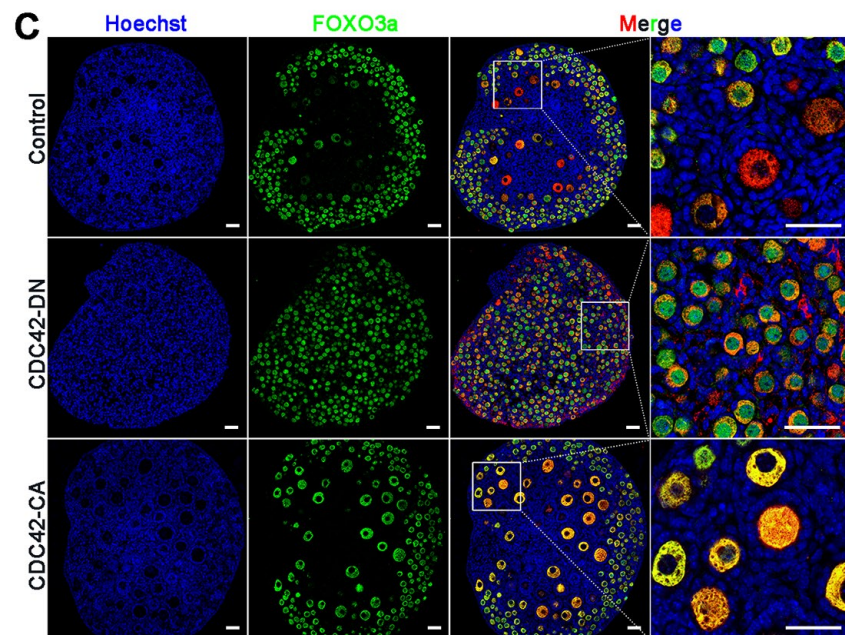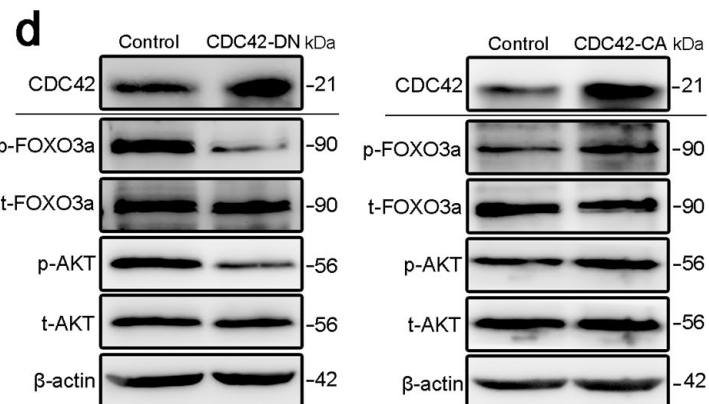

Supplement: Supplementary file 5 — Figure S5. Transfection of CDC42-DN (CDC42T17N) or CDC42-CA (CDC42Q61L) lentivirus constructs disrupts the activation of primordial follicle through regulating PI3K signaling in neonatal ovaries. (a) The ovaries at 1 dpp were transfected with empty lentivirus, lentiviral construct expressing CDC42-DN or CDC42-CA for 2 days in vitro. Green fluorescence from the GFP reporter was observed in ovaries following 2 days of lentiviral infection indicating a satisfactory efficiency of lentivirus transfection. (b) Oocyte counting results showed a comparable number of total oocytes in control, CDC42-DN, and CDC42-CA ovaries after 6 days of treatment. (c and d) Transfection of CDC42-DN and CDC42-CA affected the activity of PI3K signaling in ovaries. CDC42-DN weakened the nucleus-cytoplasm shuttle of FOXO3a (green fluorescence) in the oocytes, whereas CDC42-CA increased the cytoplasmic localization of FOXO3a in transfected ovaries. Oocytes were stained with DDX4 (red). Nuclei were dyed with a Hoechst counter-stain (blue) (c). Immunoblotting results showed that the phosphorylation of FOXO3a and AKT was decreased in CDC42-DN ovaries but increased in CDC42-CA ovaries. Levels of total FOXO3a, AKT, and β-actin were used as internal controls (d). The experiments were repeated at least three times, and representative images are shown. Scale bars, 50 μm. (PDF 842 kb) [file 12915_2018_541_MOESM5_ESM.pdf]

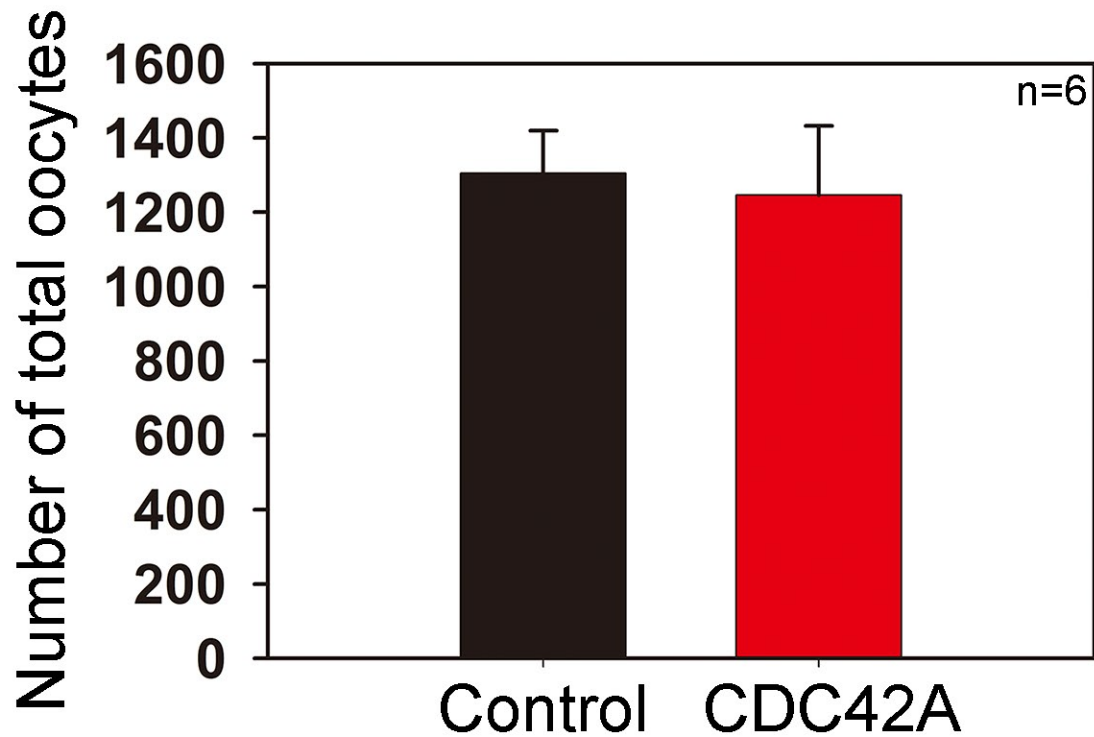

Supplement: Supplementary file 6 — Figure S6. The survival and development of ovarian follicles were not affected by CDC42 activator treatment. Ovaries were collected at 6 dpp and cultured with or without CDC42 activator for 30 min then transplanted under kidney capsules of ovariectomized adult hosts. After 2 weeks of transplantation, the number of total oocytes in ovaries was quantified. CDC42 activator-treated ovaries showed a comparable number of total oocytes compared to the control (Additional file 10: Individual data values). (PDF 464 kb) [file 12915_2018_541_MOESM6_ESM.pdf]
