# Supplementary material for: CDC42 controls the activation of primordial follicles by regulating PI3K signaling in mouse oocytes
Source: BMC Biol. 2018 Jul 5;16:73. doi: 10.1186/s12915-018-0541-4 (PMC6033292; doi:10.1186/s12915-018-0541-4)
Supplement: Supplementary file 7 — Table S1. Follicle counting results. (DOCX 15 kb) [file 12915_2018_541_MOESM7_ESM.docx]

Table S1 Follicle counting results showed the distribution of follicles after treatment with and without CDC42 Activator in 6 dpp and 35 dpp ovaries.

| Ovary | Stage of follicles | CDC42A | Control |
| --- | --- | --- | --- |
| 6 dpp | primordial | 31.08 ± 4.14% | 54.23 ± 3.83% |
|  | primary | 29.00 ± 3.75% | 17.69 ± 5.08% |
|  | secondary | 32.95 ± 2.12% | 23.80 ± 3.75% |
|  | antral | 6.97 ± 1.40% | 4.28 ± 0.70% |
| 35 dpp | primordial | 39.84 ± 4.08% | 61.42 ± 2.24% |
|  | primary | 46.99 ± 5.20% | 31.37 ± 1.49% |
|  | secondary | 11.18 ± 3.00% | 6.28 ± 1.01% |
|  | antral | 1.98 ± 0.94% | 0.93 ± 0.42% |
