# Supplementary material for: CDC42 controls the activation of primordial follicles by regulating PI3K signaling in mouse oocytes
Source: BMC Biol. 2018 Jul 5;16:73. doi: 10.1186/s12915-018-0541-4 (PMC6033292; doi:10.1186/s12915-018-0541-4)
Supplement: Supplementary file 8 — Table S2. List of primers used in qRT-PCR. (DOCX 14 kb) [file 12915_2018_541_MOESM8_ESM.docx]

Table S2 List of primers used in qRT-PCR

| Genes | Forwards (5’……3’) | | Backwards (5’……3’) |
| --- | --- | --- | --- |
| *Cdc42* | CCCATCGGAATATGTACCAACTG | CGGTCGTAGTCTGTCATAATCCT | |
| *Pten* | TGGATTCGACTTAGACTTGACCT | GCGGTGTCATAATGTCTCTCAG | |
