# Supplementary material for: CDC42 controls the activation of primordial follicles by regulating PI3K signaling in mouse oocytes
Source: BMC Biol. 2018 Jul 5;16:73. doi: 10.1186/s12915-018-0541-4 (PMC6033292; doi:10.1186/s12915-018-0541-4)
Supplement: Supplementary file 9 — Table S3. List of antibodies. (DOCX 14 kb) [file 12915_2018_541_MOESM9_ESM.docx]

Table S3 List of antibodies

| **Antibody** | **Catalogue** | **Batch number** | **RRID number** | **Company** |
| --- | --- | --- | --- | --- |
| CDC42 | Ab64533 | GR3184317-1 | AB_1310067 | Abcam |
| PTEN | 9188 | 6 | AB_2253290 | Cell Signaling Technologies |
| p110β | Ab151549 | GR3179297-1 |  | Abcam |
| DDX4 | Ab13840 | GR3218256-1 | AB_443012 | Abcam |
| DDX4 | Ab27591 | GR290112-1 | AB_11139638 | Abcam |
| AKT | AA326 | 1 |  | Beyotime Biotechnology |
| p-AKT | AA331 | 1 |  | Beyotime Biotechnology |
| FOXO3a | 12829 | 5 | AB_2636990 | Cell Signaling Technologies |
| FOXO3a | Sc-11351 | G1212 | AB_640610 | Santa Cruz |
| p-FOXO3a | Sc-12357 | I2110 | AB_653226 | Santa Cruz |
| β-actin | CW0096B | 151511K |  | CW Biotechnology |
| CDC42 | Ab187643 | GR235757-15 |  | Abcam |
| p110β | Ab1678 | GR3182212-1 | AB_302513 | Abcam |
| IgG | Sc-2763 | K0615 | AB_737189 | Santa Cruz |
| IgG | Sc-2762 | F1915 | AB_737179 | Santa Cruz |
